# Sewer microbiomes shape microbial community composition and dynamics of wastewater treatment plants

**DOI:** 10.1093/ismejo/wraf213

**Published:** 2025-09-22

**Authors:** Marie Riisgaard-Jensen, Rodrigo Maia Valença, Miriam Peces, Per Halkjær Nielsen

**Affiliations:** Center for Microbial Communities, Department of Chemistry and Bioscience, Aalborg University, 9220 Aalborg, Denmark; Center for Microbial Communities, Department of Chemistry and Bioscience, Aalborg University, 9220 Aalborg, Denmark; Center for Microbial Communities, Department of Chemistry and Bioscience, Aalborg University, 9220 Aalborg, Denmark; Center for Microbial Communities, Department of Chemistry and Bioscience, Aalborg University, 9220 Aalborg, Denmark

**Keywords:** sewer microbiome, activated sludge, wastewater treatment plants, microbial community assembly, microbial immigration, microbial ecology, biofilms

## Abstract

The link between the sewer microbiome and microbial communities in activated sludge wastewater treatment plants is currently poorly understood despite the systems being directly interconnected. Microbial immigration from wastewater has been identified as a key factor determining activated sludge community assembly. Here, we present the first comprehensive study of the sewer microbiome and hypothesize that the microbiome harbors process-critical activated sludge microbes and is thus critical for activated sludge community assembly and performance. We integrated species-level microbial analyses of biofilm, sediment, and sewer wastewater in domestic gravity and pressure sewers in Aalborg, Denmark, with samples from influent wastewater and activated sludge from two downstream wastewater treatment plants. By tracing the sources of incoming bacteria and determining their growth fate in the activated sludge, we confirmed that most activated sludge process-critical bacteria were part of the sewer microbiome. Within the sewer system, a gradient was observed, from dominance of gut bacteria in the wastewater upstream to the prevalence of biofilm and sediment bacteria downstream at the wastewater treatment plant inlet, with the relative ratio strongly affected by rain events. A holistic understanding of the sewer system and activated sludge is essential, as sewers hold massive amounts of active biomass serving as a major microbial source for community composition and dynamics in wastewater treatment plants. Sewer systems should be recognized as a crucial environmental filtration step, and the sewer microbiome as an important source for activated sludge, helping to explain the observed regional and global differences in activated sludge community structure.

## Introduction

Microbial immigration from incoming wastewater has been highlighted as a key factor in controlling microbial community assembly in municipal activated sludge (AS) wastewater treatment plants (WWTPs) [1–4]. AS is the principal process for the removal of pollutants and recovery of resources in WWTPs worldwide and it is essential to understand the mechanisms behind the assembly of the inherent microbial community to improve plant stability and performance. Thus, insights into the sources of the process-critical taxa in WWTPs—such as the species involved in nitrification, denitrification, or poor floc formation—are crucial, but they are currently lacking despite their obvious importance.

**Figure 1:**
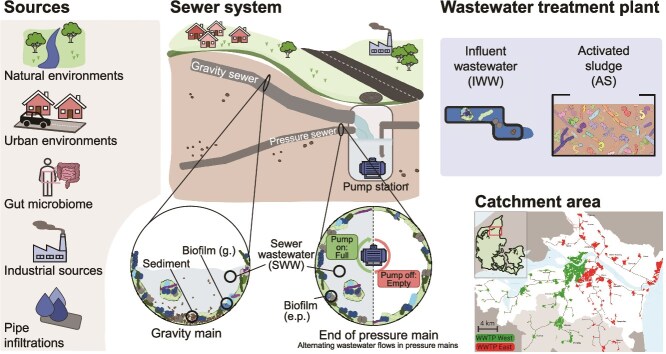
Graphical illustration of the system. Water and wastewater from different sources are introduced into the sewer system and transported through gravity and pressure mains to the wastewater treatment plant. Different samples were collected in the sewer system. Gravity (g.) mains: biofilm (g.), sediment, and sewer wastewater (SWW). End-of-pressure (e.p.) mains: biofilm (e.p.) and SWW. The bottom-right corner is a map of the catchment area (adapted from Aalborg municipality [29]).

The sewer system is a complex underground network of pipes designed to collect and convey pollutants to WWTPs from a large catchment area. The sources of microorganisms entering sewers are manifold: private households (including the human gut microbiome), urban runoff, discharges from various industries, and infiltration. In addition, there are the indigenous microbes growing within sewer systems in biofilms and sediments (Fig. 1). Together, these sources shape the incoming wastewater to WWTPs, leading to a diverse and transient microbial composition [5]. The relative contributions of each source to the influent wastewater community are mostly unknown and may vary among catchment areas.

The sewer microbiome has been defined in different ways: bacteria only in the bulk wastewater phase [6, 7], bacteria specifically in sewer biofilms [8, 9], and bacteria only in sewer sediments [10, 11]. Here, we define the sewer microbiome as the bacteria growing in sewer biofilms and sediments. These communities are thought to be actively maintained in self-cleaning sewer pipes, where the wastewater flow continuously detaches and suspends biomaterial while providing nutrients [12]. The sewer microbiome appears to be highly coherent across the world, despite differences in geography, pipe design, sewer operation, and wastewater composition. The commonly found genera are *Acidovorax*, *Acinetobacter*, *Aeromonas*, *Arcobacter*, *Cloacibacterium*, and *Trichococcus* [8, 13–16]. This suggests that the sewer infrastructure holds an overall consistent environment where these bacteria grow despite strongly fluctuating conditions. There are, however, also observed community differences, e.g. in sewer sediments depending on the catchment area and sediment depth [10, 11], in sewer biofilms depending on the position relative to the water level [17], along the length of the pipe and correlated with the availability of substrate [11, 18], and between different sewer types, such as gravity and pressurized systems [15].

Most research on sewer microorganisms has focused on the taxa involved in the deterioration of sewer pipes, pollution with pathogenic bacteria to environments, or the spread of antibiotic-resistance genes [9, 19–21]. However, broader insights into the sewer microbiome’s ecological role are limited, partly due to inconsistent sampling across compartments (e.g. wastewater, biofilms, sediments) and few studies considering spatial and temporal variation [7, 22]. The sewer microbiome may represent a previously overlooked source of process-critical microorganisms in AS plants and thus determine the AS community assembly. In a recent study across 84 WWTPs in Denmark, we observed species-level differences in AS communities by using the ecosystem-specific MiDAS reference database [23, 24]. These differences included functionally important AS taxa and were similarly evident in the taxa present in the incoming wastewater, which shaped the observed community variations. Therefore, greater attention should be given to the factors shaping influent wastewater (IWW).

IWW contains a mixture of taxa from various sources, including the sewer microbiome. The composition shifts in response to both diurnal and seasonal changes as well as geographical variations [5, 7, 13, 25, 26]. An obvious source of the IWW composition is gut bacteria, and highly variable abundances of gut bacteria have been reported: ranging from <1%–51% of the total abundance in IWW to 8%–66% in wastewater taken directly upstream in sewer pipes [7, 13, 27, 28]. The biomass introduced into AS plants from sewer systems is substantial, with an average of 5% of the AS biomass reported to reach the plants daily [2]. Most of the biomass entering WWTPs dies off, and a fraction of this biomass likely originates from the human gut and sewer microbiome. Many of the abundant genera commonly found in sewers and IWW, such as *Acidovorax*, *Aeromonas*, *Arcobacter*, *Cloacibacterium*, and *Trichococcus*, have been observed to die off in AS plants and are only present due to continuous immigration from IWW [2]. Only a small fraction of the bacteria present in IWW grows in the AS, defined as the “process-critical bacteria” [2]. In Danish WWTPs, these growing taxa accounted for only 1%–3% of the total read abundance in IWW (300–400 taxa), including the key microbes involved in nutrient removal, such as nitrifiers, denitrifiers, and polyphosphate-accumulating bacteria.

The sources of these key taxa are critical to understanding the assembly of the AS microbiome [24]. Sewer systems, which include gravity mains with dynamic oxic–anoxic conditions and primarily anoxic pressure mains with varying levels of organic content, may provide favorable growth conditions for many AS process-critical taxa. However, this relationship remains poorly understood. Additionally, many sewer systems also convey rainwater, meaning that rain events could significantly impact these growth conditions.

In this study, we aimed to establish a comprehensive overview of all microbes in domestic IWW, identify their potential sources, and characterize their fate in AS plants. We have a special focus on the sewer microbiome, which encompasses bacteria growing in biofilms and sediments, and hypothesize that the sewer microbiomes harbor many process-critical AS microbes, thus largely determining the community assembly and function of the AS plants.

## Materials and methods

### Sewer system investigated

The sewer system in Aalborg Municipality, Denmark, consisted in 2023 of 2216 km of main and 460 km of branch pipes; 222 km of the main pipes were pressure pipes. The distribution of separated (no rain) and combined sewer lines was 72.3% separated lines. In total, 10.4 million m^3^/year of wastewater was registered, with the capacity of wastewater treatment in Aalborg Municipality being 430 000 PE, treated at either Aalborg East (AAE) WWTP (25%) or Aalborg West (AAW) WWTP (75%) [29–31]. Samples from different habitats were collected in the sewer system in gravity (g.) pipes and endpoints-of-pressure (e.p.) pipes (2022–3) and at the WWTPs (from 2019 to 2022) (Supplementary Fig. S1). All the sewer samples were collected upstream of the WWTPs, within residential areas in close proximity to households (Supplementary Fig. S2). From gravity pipes sediment, biofilm (g.), and sewer wastewater (SWW) were collected, and from end-of-pressure pipes biofilm (e.p.) and SWW were collected. Gravity pipes have a fairly consistent flow of wastewater, whereas in pressure lines wastewater was sampled when wastewater was pumped through the pipe. At the WWTPs, IWW after primary settling and AS from the oxic process tank were collected (Fig. 1). IWW was sampled after primary settling due to consistent data availability across sites and time points. Primary settling can alter the relative read abundance of some bacteria; however, these changes are typically minor, and the overall community composition (presence/absence) and seasonal patterns tend to remain stable [32]. Additionally, while IWW and AS samples were collected in different years than the sewer samples, AS communities are known to be temporally stable, with recurring seasonal patterns [33].

### Sampling

Samples were gathered four times over 1 year from nine distinct locations within Aalborg municipality, collecting primarily municipal wastewater originating from combined (*n* = 6) or separated (*n* = 3) sewer systems. Collection points included manholes along gravity pipes (*n* = 7) and endpoints of pressure lines (*n* = 2). At each location, SWW, biofilm, and sediment were collected. Not all habitats could be consistently collected at every spot as adverse weather conditions, particularly heavy rainfall, hindered the collection of specific habitats. The total number of samples included has been summarized in Supplementary Table S1 and more details can be found in Supplementary File 1. All the samples were obtained from ground level using an extending pole to reach the sewer. SWW samples were taken as grab samples of ~500 ml each. Biofilm was collected by scraping the bottom half of the pipe with clean steel wool, gathering 5–10 ml of biofilm per sampling. Sediments were acquired using a hollow spoon, collecting 50–100 ml of sediment per sampling. All the samples were stored at −20°C. All the sample locations led to either the AAW or the AAE WWTP, and samples from their IWW and AS were included in the study. IWW samples were 50 ml subsamples from 24-hour flow-proportional samplers, while AS samples were 2 ml subsamples from 50 ml grab samples taken from aeration tanks (2019–22). Rainfall data for Aalborg was sourced from the Danish Meteorological Institute.

### Amplicon sequencing workflow

A total of 235 samples were subjected to 16S rRNA gene sequencing (Supplementary Table S1). Before DNA extraction, samples were thawed, vortexed, and homogenized using an overhead stirrer (Heidolph RZR 2020, Germany) set to the second gear at speed nine. The homogenization process involved moving the sample inside the tissue grinder 10 times from top to bottom. After homogenization, IWW and SWW samples underwent vacuum filtration through a 0.2 μm polycarbonate membrane supported by glass fiber filters, utilizing a DHI filtration manifold (Carbon 14 Centralen, Denmark) to immobilize cells from IWW and SWW on the surface of the membrane. Subsamples of the homogenized biofilm, sediment, and AS (biofilm: 0.5 g, sediment: 0.2 g, AS: 0.5 ml) were taken for DNA extraction and later processed using the same DNA extraction and amplicon sequencing method as previously described [2]. The V1–V3 primers used had the following sequences: 27F 5′-AGAGTTTGATCCTGGCTCAG-3′ [34]; 534R 5′-ATTACCGCGGCTGCTGG-3′ [35]. The V1–V3 region was targeted due to its broad taxonomic resolution and compatibility with existing datasets [2, 23, 24]. All the raw amplicon reads produced by the MiSeq System (Illumina) were processed using the AmpProc 5.0 workflow (https://github.com/eyashiro/AmpProc), which generated Amplicon Sequence Variants (ASVs). The ASVs were mapped to full-length ASVs from the MiDAS 5.3 database [36] using “usearch” with *-*sintax_cutoff 0.6. We selected a cutoff of 0.6, based on empirical evaluation against the commonly used threshold of 0.8, as it provided a reasonable trade-off between assignment sensitivity and specificity in this dataset (Supplementary Fig. S3B).

### Data analysis

Data analysis was performed in R v4.4.0 (https://www.R-project.org/) using RStudio 2024.04.1+748. The R packages “ampvis2” and “vegan” were used for rarefaction, distance matrices, alpha diversity measures, and Permutational Multivariate Analysis of Variance (PERMANOVA) testing with the “adonis” function [37, 38]. Scripts and data are available at https://github.com/MarieRiisgaard/2023_sewer_microbial_communities and bioproject PRJNA946374 and PRJNA1139651. Only samples with >5000 reads were included and duplicates were merged by the mean read count. For analysis of alpha diversity, samples were rarefied to 7322 reads, corresponding to the sample with the fewest reads. As the sequencing depth was much higher for IWW and AS (median: 67 710 reads), these samples were rarefied to the median read count of the sewer samples (median: 14 728 reads) for all other analyses (Supplementary Fig. S4).

### Bacterial groups

Gut bacteria were classified by mapping the 16S rRNA gene sequences from human gut bacteria databases [39, 40] to the full-length ASVs in the MiDAS database, using a >98.7% identity cutoff for species classification, in line with accepted standards in 16S rRNA gene-based microbial profiling [41, 42]. We defined process-critical bacteria as taxa that can grow in AS. Thus, process-critical bacteria include all bacteria with a positive growth rate, including, but not limited to, known functionally important groups such as nitrifiers, denitrifiers, and polyphosphate-accumulating organisms. The net growth rate was calculated based on the mass balances between IWW and AS samples that have commonly been applied in AS systems [2, 43, 44]. This approach was recently used across 84 Danish WWTPs, and the resulting growth fates groups from this study were used [24] (Supplementary File 1). The following groups were defined—growing: positive growth rate; disappearing: negative growth rate; surviving: no growth; variable: growth rate dependent on WWTP.

The core sewer taxa were defined separately for each sewer microbiome (biofilm [g.], biofilm [e.p.], and sediment). A species observed in ≥0.01% relative abundance in ≥80% of the samples was defined as strict core species, ≥50% general core, and ≥ 20% loose core [23]. A threshold of >0.01% relative abundance corresponded to >2 reads, given the median read count of 17 896 for biofilm (g.), biofilm (e.p.), and sediment samples. If a species was observed with any reads in >2 samples, it was defined as “detected in the sewer microbiome.” Bacteria classified as gut bacteria were excluded as part of the sewer core groups. The resulting core groups were merged to find the “unified core” for each species. Similarly, the abundant core genera for each habitat were defined for each sewer microbiome as genera present in ≥0.05% abundance in ≥80% of the samples. Core groups are listed in Supplementary File 1.

## Results

### The microbial communities in the sewer system and the WWTPs

Samples from different habitats (biofilms, sediments) were collected in the sewer system in gravity (g.) pipes and endpoint-of-pressure (e.p.) pipes. Samples also included SWW and at the WWTPs IWW and AS (Fig. 1). All the obtained ASVs were classified with the MiDAS reference database, and >94% of the accumulated abundance had a species match in the database. However, some were not classified due to multiple species matches in the V1–V3 16S rRNA gene region, leaving 81%–94% of the total abundance classified to species level (Supplementary Fig. S3A).

**Figure 2:**
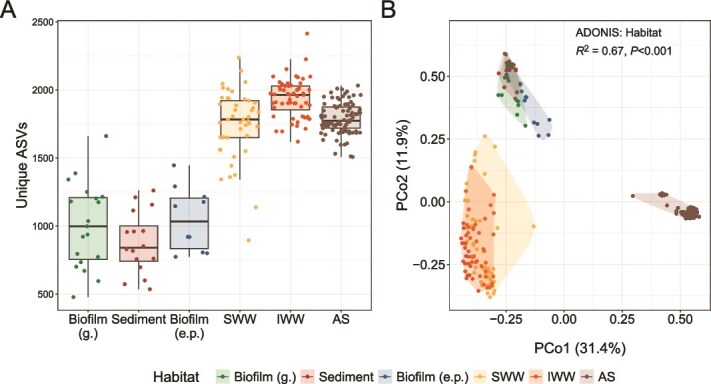
Alpha and beta diversity. Samples are grouped and colored based on habitat: biofilm (g.), biofilm (e.p.), sediment, sewer wastewater (SWW), influent wastewater (IWW), and activated sludge (AS) from AAW and AAE. (A) Number of observed ASVs (all the samples were rarefied to 7322 reads). (B) PCoA of all samples at the ASV level using Bray–Curtis distance matrix (relative abundance > 0.01%). An PERMANOVA analysis showed habitat to explain 67% (*P* < .001) of the microbial community variation.

The diversity of the microbial community was highly differentiated depending on habitat. Rarefied samples had 873–1941 different ASVs, with the highest number in IWW and the lowest in sediments (Fig. 2A). IWW, SWW, and AS all had a high diversity compared to the sewer habitats (biofilm [g.], biofilm [e.p.], sediment) and this observation was common for all used alpha diversity indexes (Supplementary Fig. S5). The lower diversity in the sewer habitats was mainly caused by a few abundant ASVs, often comprising >25% of the accumulated relative read abundance (Supplementary Fig. S6). In the wastewater, the species diversity was higher for IWW compared to SWW, likely due to the collection of microorganisms from the larger catchment area for IWW and because SWW samples were grab samples rather than 24-hour proportional samples.

Beta diversity analysis revealed strong differences in community composition between habitats (*R* [2]: 0.67, *P* < .001; Fig. 2B). SWW and IWW formed distinct clusters but were more similar to each other than to the sewer-associated habitats, which in turn clustered together. The Principal Coordinate Analysis (PCoA) components explained >40% of the sample variation, clearly showing the uniqueness of the microbial community in each cluster. One SWW sample positioned away from the main cluster was collected during a rain event and represents one of several rain-affected samples that prompted further investigation into rainfall impacts (see later). Of the 15 most abundant genera for each habitat, only *Acidovorax* and *Trichococcus* were common, and the remaining genera were mostly unique for each of the three clusters (Supplementary Fig. S7).

### Important sources of taxa in IWW are human gut microbiota and members of the sewer microbiome

**Figure 3:**
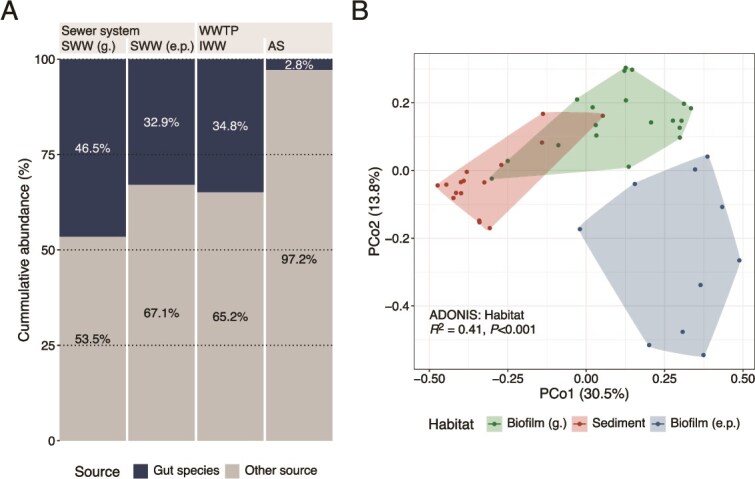
Abundance distribution of gut species (A) and beta diversity of the sewer microbiome (B). (A) Cumulative abundance of gut species with the abundance fraction shown for each group. (B) Beta diversity of the sewer microbiome with samples grouped and colored based on the habitat: Biofilm (g.), sediment, and biofilm (e.p.). The PCoA was made at the species level using the Bray–Curtis distance matrix (relative abundance > 0.01%). An PERMANOVA analysis showed habitat to explain 41% (*P* < .001) of the microbial community variation.

We investigated the potential sources of all taxa in the IWW, focusing on three main categories: the human gut, the sewer microbiome, and unknown sources, which include inputs from industries, hospitals, and other unidentified origins. Human gut bacteria, such as *Ruminococcus faecis* and *Eubacterium rectale*, accounted for a large fraction of the wastewater in the sewer system, particularly in gravity sewers (SWW [g.]), where they made up 49% of the total read abundance due to the proximity to households (Fig. 3A and Supplementary Fig. S8). At the end of pressure pipes, where water is often stored at pumping stations before transport, and further downstream in the IWW, this fraction decreased to 35%, likely due to decay during storage and transport, dilution by detached taxa from sewer biofilms and sediments, and the growth of these in the water phase.

To test our hypothesis that the sewer microbiome serves as a crucial source of process-critical bacteria in AS, we conducted a detailed analysis of its various habitats. Beta diversity analysis revealed three distinct microbial groups corresponding to specific habitat types: biofilm (g.), sediment, and biofilm (e.p.) (Fig. 3B). Habitat alone explained 41% of the community difference (PERMANOVA analysis). The exact community composition is described later. When both sediment and biofilm were obtained at the same location in gravity sewers, the overall community could be explained by habitat (*R* [2]: 0.31, *P* < .001), and sample location—across both biofilm and sediment—also had a significant effect (*R* [2]: 0.37, *P* < .001) (Supplementary Fig. S9). Additionally, several species were specific to or particularly abundant at individual locations. For example, a *Variovorax* sp. was only observed at a single site (Supplementary Fig. S10).

**Figure 4:**
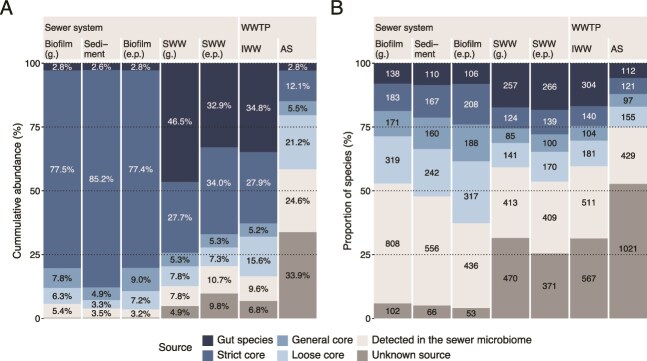
Contribution of bacteria from different sources to the IWW and AS communities. (A) Cumulative abundance of sewer microbiome unified core groups and gut bacteria for each group, with the abundance fraction shown if >2.5%. (B) Proportion of species and unclassified ASVs across habitats. Unclassified ASVs were treated as individual species.

The sewer microbiome, defined as the taxa growing in biofilms and sediments, can be challenging to characterize due to potential contamination from nongrowing taxa present in the water phase during sampling. To address this, we applied a relative abundance cutoff of 0.01% and focused on core groups, which are species consistently observed across multiple samplings and locations. This approach reduced the likelihood of including taxa originating from the water phase. Each of the three sewer microbiome habitats (biofilm [g.], sediment, and biofilm [e.p.]) contained core species specific to that environment (Supplementary File 1). To identify the unified core of the sewer microbiome, defined as core species found in any of these habitats, we combined the core species identified in each habitat, giving priority to strict core species, followed by general and then loose core species (Fig. 4). More than 90% of the relative abundance in the sewer habitats belonged to the unified strict and general core, clearly showing that most species occurred across multiple sites for a certain habitat type. A small fraction of gut bacteria (<3%) was observed; their presence likely reflected wastewater deposition during sampling in a flowing pipe rather than active growth. In SWW and IWW, the unified core bacteria of the sewer microbiome accounted for most of the remaining abundance not identified as gut bacteria. The unified loose core doubled in IWW compared to both SWW (g.) and SWW (e.p.), suggesting that during transport, more site-specific species have detached from sewer surfaces or proliferated within the SWW, contributing to the high diversity observed in the IWW.

In the sewer habitats, detected in the sewer microbiome (low-abundant species [<0.01%] detected in >1 sample in either biofilm [g.], sediment, and biofilm [e.p.]) accounted for only 3%–5% of the relative abundance but represented 30%–40% of the species diversity. As this group was tracked through the sewer system, its relative abundance increased, reaching 8%–9% in wastewater and ultimately making up 24.6% of the abundance in AS. This indicates that the sewer microbiome hosts many low-abundant species that were excluded from the sewer core communities. Consequently, it was not possible to track the source for all species and ~7% (>500 species) of the accumulated abundance in IWW and 33.9% (>1000 species) in AS originated from unknown sources (Fig. 4).

### Identifying bacterial growth fates of sewer bacteria in AS

**Figure 5:**
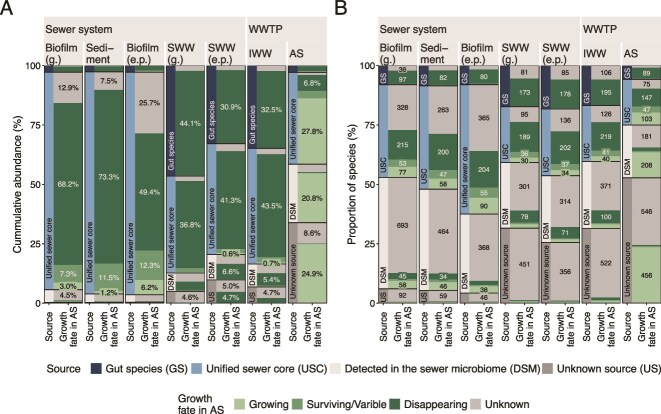
Species classified by their growth fate in AS and source environment. (A) Cumulative abundance of sewer microbiome core groups (unified strict, general, and loose cores were merged) and gut bacteria with the abundance fraction (if >4% or growing and >0.5%) shown for each group. (B) Proportion of species and unclassified ASVs across habitats. Unclassified ASVs were treated as individual species. Absolute numbers of distinct species were annotated when they represented >2% of the total species count. The unknown source category represents species detected in AS and IWW that were not assigned to any of the defined upstream groups.

We tracked the bacteria from gut and sewer habitats (sewer microbiome) to establish their fate in the WWTP. We were particularly interested in the sources of the AS process-critical (growing) bacteria, and bacteria in each source group (Fig. 4) were classified by growth fate in AS (Fig. 5). Essentially all the AS taxa were also found in the IWW but roughly 10% of the cumulative abundance was not assigned to a growth fate as the abundances of these were too low in the IWW and AS. One of the largest fractions in SWW and IWW, the gut bacteria, disappeared in the AS, likely due to the different growth conditions in the AS and human gut.

The unified sewer microbiome had both taxa disappearing and growing in the AS. The growth fate of each core group was also analyzed separately (Supplementary Fig. S11). The bacteria disappearing in AS included two very abundant species, *Trichococcus* midas_s_4 and *Acidovorax* midas_s_2077. These species accounted for 45% of the relative read abundance in biofilm (g.) and sediment, and 25% in biofilm (e.p.), whereas their contributions to IWW and AS were <15% and 4%, respectively (Supplementary Fig. S12).

The core taxa in the sewer habitats growing in AS were present in low relative abundance across sediment and biofilms, making up only 1.2%–6.2%, but were diverse, comprising 58–90 distinct species (Fig. 5). These species eventually accounted for 27.8% of the relative abundance in AS, showing that many process-critical bacteria thriving in AS originated from biofilm and sediment environments, including most of the abundant taxa (Supplementary Table 2).

We examined the group of low-abundance species (<0.01%) detected in multiple sewer habitat samples (detected in the sewer microbiome; Fig. 5). Despite being highly diverse, with over 350 species, this group accounted for <3% of the cumulative abundance in sewer habitats. However, in AS they represented 20.8% of the total abundance, >200 of the species were growing in AS, and included important process-critical species, such as *Ca.* Microthrix parvicella, *Azonexus phosphoritropha*, and *Ca.* Competibacter species. Species within this group exhibited varied patterns of detection (Supplementary Fig. S13A). *Ca.* M. parvicella and *A. phosphoritropha* were primarily found in wastewater, suggesting their main origin lies outside of the sewer biofilm and sediments sampled in this study. Other species, such as additional *Azonexus* members and *Ca.* Competibacter and *Hyphomicrobium* spp., were consistently detected at low abundance in biofilms and sediments across most samples within each habitat, with some of the species occurring at only one or a few sites. Overall, this pattern suggests that these species are likely residents of the sewer microbiome despite their low abundance.

As mentioned, we were not able to identify the source for a fraction of the growing species in AS (“unknown source,” Fig. 5). Looking only at the growing fraction in AS, they represented 25% relative abundance in AS (~350 species). The most abundant bacteria in this group was a *Ca.* Villigracillis species (1%–1.2% relative abundance in AS) (Supplementary Fig. S13B). They were consistently present in the IWW, but we did not find their sources, which could potentially be linked to specific industries or unsampled areas of the sewer network, or the upper, drier surfaces of gravity pipes, highlighting the need for further investigation into these contributors.

### Sewer microbiome habitats host specialized communities

**Figure 6:**
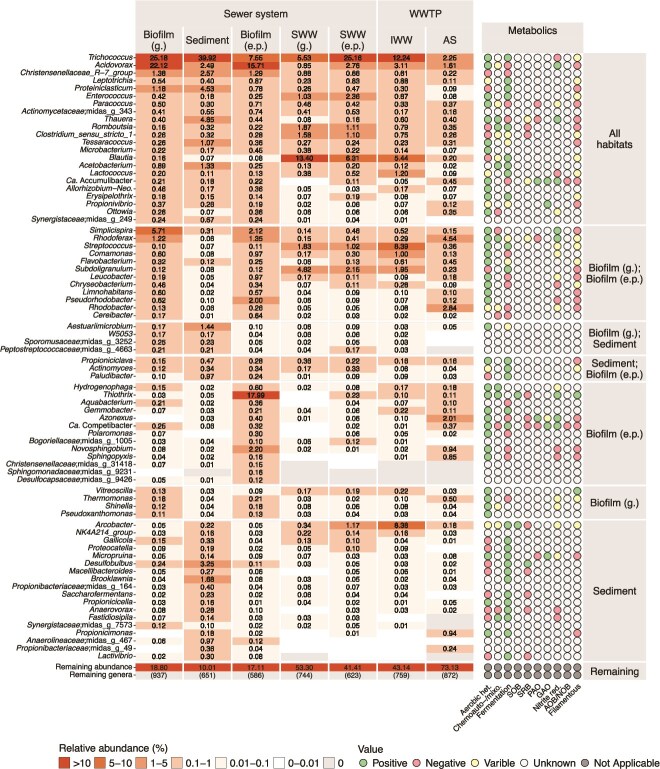
Abundance of core genera in biofilm (g), sediment, and biofilm (e.p.). The abundant core was defined as genera with a relative abundance ≥ 0.05% in ≥80% of the samples for each habitat. The right-hand panel shows in which sewer habitat a given genus was a part of the abundant core. Metabolic potentials were taken from the MiDAS field guide (06-18-2024). “Filamentous” is not a metabolic property but refers to the morphology of the genus. The median abundance is shown with two decimals if ≥0.01%. “Remaining abundance” and “remaining genera” refer to the remaining abundance or number of genera, respectively. Only classified genera are included, with family names provided for genera classified with MiDAS placeholder names.

As previously described, the three sewer habitats harbored distinct communities (Fig. 3B). These habitats contained a total of 79 core genera, which together accounted for over 80% of the total abundance (Fig. 6). Of these, 22 were common to all the habitats, whereas 4, 14, and 18 genera were unique for biofilm (g.), biofilm (e.p.), and sediments, respectively. The metabolic potential of these abundant genera (where known) reflected the conditions within different sewer environments. End-of-pressure pipes with intermittent flow and gravity pipes both exhibit dynamic oxic–anoxic conditions and fluctuating levels of organics. Gravity pipe sediments and biofilms may be thick and stratified, containing both oxic and anoxic layers. In line with this, the dominant taxa in these habitats were primarily aerobic heterotrophs and/or fermentative bacteria, whereas genera unique to sediment environments were often strictly fermentative.

All the core genera were fast-growing chemoheterotrophic well suited to nutrient-rich sewers. No core genera were solely chemoautotrophic/mixotrophic, such as nitrifiers. Genera unique for the biofilm (g.) and biofilm (e.p.) (both shared and unique for each) were predominantly aerobic heterotrophs and genera unique for sediments were strict or facultative anaerobes. In sediments and biofilms, both sulfate-reducing bacteria, such as *Desulfobulbus*, and sulfur-oxidizing bacteria, such as *Arcobacter*, were present and appeared adapted to the dynamic conditions in these environments [45]. These genera are also linked to sewer corrosion [46]. *Thiothrix* was particularly abundant in biofilm (e.p.) and coincided with a strong smell of hydrogen sulfide during sampling.

Important functional groups in AS were present in the core genera of the sewer microbiome, including polyphosphate-accumulating organisms (PAOs), glycogen-accumulation organisms (GAOs), filamentous bacteria, and potential denitrifiers. PAOs were observed in all sewer environments, with *Azonexus* and *Ca.* Accumulibacter being core genera in all sewer environments or biofilm (e.p.), respectively, often at relative abundances similar to those seen in WWTPs. The GAOs *Ca.* Competibacter, *Propionivibrio*, and *Micropruina* were also abundant. Potential denitrifiers were observed in all habitats with *Acidovorax* and *Thauera* being most abundant. Many filamentous bacteria important for floc formation in AS were also part of the abundant core, with *Thiothrix* being especially prevalent in biofilm (e.p.).

We searched for the nitrifiers *Nitrospira*, *Nitrotoga*, and *Nitrosomonas*, which are essential for nitrogen removal in WWTPs, as well as the filamentous genera *Ca.* Microthrix and *Ca.* Amarolinea, which are linked to foaming and bulking in AS. All of these were classified as “Detected” in the sewer microbiome (Figs 4 and 5) due to their low but consistent presence (>0.05%) across sites (Supplementary Fig. S14). They were more prevalent in SWW, particularly in IWW, indicating that these bacteria are transported through the sewer system; however, their dominant niche within the sewer system remains to be determined.

### Influent wastewater resembles the sewer microbiome after rain

Most sewer catchments contain sections with combined sewers, where rainwater enters the systems. The rainfall increases the flow in gravity sewers, affecting water levels, shear stress, temperature, and both organic and oxygen levels. As a result, rain events can significantly influence sewer systems and, consequently, the microbial composition of IWW and AS plants. At the microbial community level, this effect remains unexplored, and given Aalborg's numerous combined sewers, we aimed to investigate it further.

**Figure 7:**
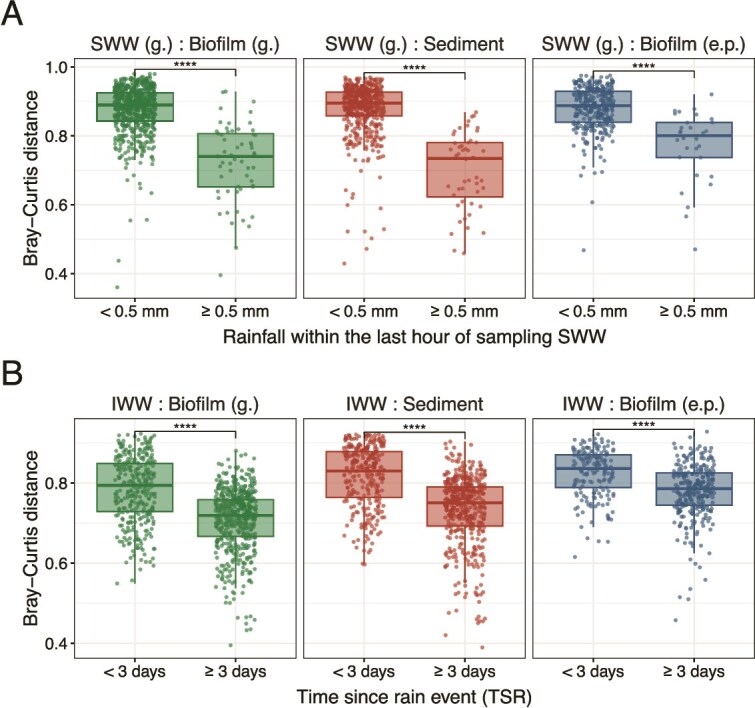
Influence of rainfall on the microbial communities in SWW from combined gravity pipes and IWW from Aalborg West WWTP. (A) Bray–Curtis distances between SWW and sewer environments depending on the rainfall within an hour before sampling (≥0.5 mm). (B) Bray–Curtis distances between IWW and sewer environments depending on time since rain event (TSR), defined for IWW samples as the number of consecutive days with <2 mm of rainfall prior to sampling. Samples are grouped based on TSR ≥ 3 days. In both (A) and (B), *t*-tests were performed, and ^****^ denotes that *P* < .0001.

The effect of rainfall on the microbial composition of SWW was striking. We compared samples taken during dry and rainy periods over 14 months (with precipitation recorded in Aalborg municipality within 1 hour before sampling) and observed that rain caused bacteria from the sewer microbiome (biofilms and sediment) to detach and enter the SWW, significantly reducing the Bray–Curtis (BC) distance between the SWW and biofilm/sediment samples (Fig. 7A). Consequently, the sewer unified core bacteria had a higher relative abundance in the rainy SWW samples, whereas the gut bacteria were less abundant (Supplementary  Fig. S15).

We wanted to investigate the effect of “the first flush.” It is well described that a heavy rain event significantly changes basic water quality parameters such as total suspended solids, chemical oxygen demand (COD), and indicator bacteria [47–49]. We hypothesized that a heavy rain event would flush biofilm and sediments toward the WWTP, shifting the IWW taxa composition toward the sewer microbiome. The extent would, however, depend on the amount of biomass built up in the sewer systems since the last rain event. To quantify this, we introduced the concept of “time since rain” (TSR), defined as the number of consecutive days with <2 mm of rainfall. Two days after a day with a rain event (rain event: ≥2 mm precipitation in a single day), the TSR resets to zero. Two days were chosen to account for both the 24-hour sample collection period and the wastewater accumulation following rainfall (illustrated in Supplementary Fig. S16).

A simple linear model was used to analyze the BC distance between IWW samples and sediment, biofilm (g.), and biofilm (e.p.) samples in relation to TSR. Although the model was based on AAW samples, the same trends were observed for AAE (Supplementary Fig. S17). As the TSR increased, BC distances decreased, supporting our hypothesis that longer dry periods allowed for more bacterial growth in the sewers, increasing the suspension of sewer bacteria into SWW after a rain event; consequently making the IWW more similar to the sewer microbiome. A sharp drop in BC distance was observed around a TSR of 3 days, prompting us to group BC distances based on this threshold (Fig. 7B). This highlights that immediately following a rain event, microbial communities in the IWW and the sewer microbiome were most dissimilar, as most bacterial biomass had already been flushed to the WWTP during the rain event.

## Discussion

**Figure 8:**
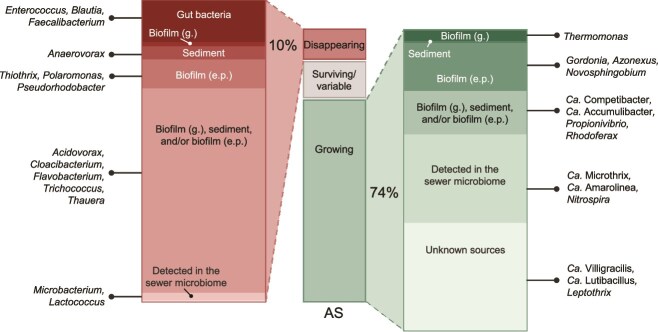
Overview of sources of bacteria in AS depending on their growth fate. The central bar represents the cumulative abundance of bacterial species, summarized by their growth fate in AS. On each side, the disappearing (left panel) and growing (right panel) fractions are further broken down by the source environment, including biofilm (g.), biofilm (e.p.), sediments, and gut bacteria, detected in the sewer microbiome and unknown sources. Examples of genera with species in each source category are shown for both disappearing and growing fractions.

The biomass introduced from municipal sewer lines into biological wastewater treatment plants is substantial, accounting daily for 5% or more of the total AS biomass. However, its role in AS community assembly has largely been overlooked. When considered, the emphasis has primarily been on the abundant taxa in IWW [50, 51]. Yet, as demonstrated in our previous research, the low-abundance taxa in IWW are crucial for AS processes and all process-critical taxa must be continuously transported with the incoming wastewater to maintain the populations in the AS [1, 2, 24]. Sewers are often described as bioreactors that modify wastewater during transport, but they also function as an environmental filter before the AS plants, reshaping the microbial composition and increasing microbial diversity [8, 12]. In doing so, the sewer system plays a crucial role in continuously seeding the microbial community of AS systems (Fig. 8).

The primary source of taxa in IWW was the human gut and the sewer microbiome. Additionally, we observed taxa from unknown sources and these taxa may vary between sewer systems, contributing to differences in microbial communities across catchment areas and, consequently, to variations in AS community composition [24]. Our study included only nine sampling locations across an ~2500 km sewer network, representing a small fraction of the system. Sampling location was a significant explanatory variable across both sediment and biofilm samples in gravity sewers, with some sites hosting distinct, site-specific taxa. A distance–decay relationship was similarly reported in sewer biofilms from a Chinese city [52]. To improve source attribution and fully capture community variation, future studies should focus on a broader spatial coverage, including major transport lines, industrial outflows, and less accessible regions of the sewer.

We confirmed our hypothesis: the sewer microbiome serves as a source of process-critical taxa in AS plants. Although the sewer microbiome has been defined in various ways, we focused on taxa growing in sewer biofilms and sediments, which clearly demonstrated that this microbiome supplies most process-critical bacteria. Many process-critical taxa in AS are adapted to dynamic conditions of electron acceptors and donors (e.g. *Ca.* Accumulibacter, *Axonexus*, and *Ca.* Competibacter), and they experience very similar conditions in the sewer system. In sewers, nutrient and oxygen levels fluctuate dynamically, driven by daily and seasonal variations [12, 27]. Thus, the feast–famine condition promoted in AS occurs similarly in sewer pipes selecting bacteria suited for this niche.

Nitrifiers were consistently present in the IWW and classified as “Detected” in the sewer microbiome. However, they were more common in the wastewater phase, and their dominant niche within the sewer system remains to be determined. The rich organic content of the sewer habitats sampled in this study likely outcompetes slow-growing autotrophic nitrifiers, favoring heterotrophs, which were indeed found to dominate the sewer microbiome. These heterotrophs, often facultative anaerobes able to survive fluctuating oxygen levels, included highly abundant genera such as *Trichococcus* and *Acidovorax* with relative abundances up to 41% [14, 16]. Although thriving in sewers, they do not grow in AS. However, due to continuous immigration they are present in abundances of 1%–3% across Danish and global AS plants and are recorded as core members of the AS community [23, 24]. Their activity during decay in AS is unknown and they might be unrelated to AS performance. Similarly, human gut bacteria were effectively reduced during transport in the sewer and ultimately reduced from 49% relative abundance in SWW (g.) to <3% in the AS.

Rain events significantly impacted the microbial composition in the sewers and IWW, largely due to the prevalence of combined sewer lines. Under dry conditions, we observed a shift in bacterial composition during transportation: SWW near households was dominated by gut bacteria, and as it reached the WWTP as IWW, biofilm and sediment bacteria (sewer microbiome) became more prevalent. This shift was amplified during rain, caused by the detachment and suspension of biofilms and sediments. The effect was especially pronounced after a dry period followed by heavy rainfall (first flush), as the amount of sewer biomass present depended on the time available for growth.

First flush events can introduce significant biomass to WWTPs. Sewer biofilm roughly contains ~10 g_COD_/m^2^ of cell biomass [12]. In the gravity sewers of Aalborg Municipality, the system holds roughly 4.2 g_COD_ cell biomass per meter of pipeline, totaling >10 000 kg_COD_ of cell biomass across all gravity sewers (Supplementary results). In comparison, the AAW WWTP received on average 30 000 kg_COD_/day (2015–20). Assuming that 10% of the influent organic content (measured as COD) is cell biomass [53], this corresponds to a daily input of 3000 kg_COD_ of cell biomass, equivalent to an average of 14% of the AS biomass per day before or 7% after primary settling. During heavy rainfall, substantial amounts of sewer biomass are transported to the WWTP, which explains the significant shift toward a higher presence of sewer microbiome bacteria in both IWW and SWW during rain.

Geographical variations in AS microbial communities have been linked to immigration from incoming IWW, which plays a key role in shaping community assembly in WWTPs [13, 23, 24]. In this study, we expanded our analysis beyond IWW to investigate the sewer microbiome, using Aalborg Municipality as a case study to trace the origins of many process-critical species in AS. This approach provides a deeper understanding of community assembly in both IWW and AS. Key functional bacterial groups were more abundant in the sewer microbiome than in the wastewater, clearly highlighting the sewer as a major source of AS process-critical bacteria. In conclusion, sewer systems should be recognized as an environmental filtration step, selectively sorting and introducing bacterial species based on their ability to grow within sewer environments. The observed differences in AS microbial structure across global WWTPs underscore the need for biogeographical research, particularly regional studies on sewer catchment areas [13, 23, 54]. As sewer infrastructure is difficult to modify, knowledge of local microbial sources and immigration patterns may allow plant operators to anticipate the arrival of unwanted taxa and take preventive action. Additionally, differences in the presence of process-critical organisms such as nitrifiers or filamentous bacteria in AS plants with the same design may not be explained by differences in operations as commonly believed, but simply by differences in immigration patterns. Over time, integrating sewer microbiome data into WWTP management frameworks could support more predictive and resilient treatment processes.

## Supplementary Material

Supplementary_material_wraf213

## Data Availability

Amplicon sequencing data are publicly available at the National Center for Biotechnology Information (NCBI) under project accession numbers PRJNA946374 and PRJNA113965. R scripts and related data are available at https://github.com/MarieRiisgaard/2023_sewer_microbial_communities
