# Supplementary material for: Sewer microbiomes shape microbial community composition and dynamics of wastewater treatment plants
Source: ISME J. 2025 Sep 22;19(1):wraf213. doi: 10.1093/ismejo/wraf213 (PMC12516951; doi:10.1093/ismejo/wraf213)
Supplement: Supplementary_material_wraf213 [file supplementary_material_wraf213.pdf]

Supplementary results

Sewer microbiomes shape microbial community composition and dynamics of wastewater treatment plants

Marie Riisgaard-Jensen, Rodrigo Maia Valença, Miriam Peces, and Per Halkjær Nielsen

Center for Microbial Communities, Department of Chemistry and Bioscience, Aalborg University, 9220 Aalborg, Denmark

Biomass in the sewer system

To evaluate the relevance of the sewer microbiome in terms of biomass the approximate amount of biofilm from gravity sewers in Aalborg Municipality was calculated. In 2023, the sewer system in Aalborg Municipality, Denmark consisted of 2216 km of main pipes and 460 km of branch pipes. 222 km of the main pipes were pressure pipes. The distribution of separated and combined sewer lines was 72.3% separated lines. The details about the pipe diameters are given in Table S1. When calculating the amount of biofilm, it must be considered whether it is a gravity or pressure pipe, and whether it is from separated or combined systems. Calculations from pressure pipes were not included. In gravity sewers, the wetted perimeter - thus the area where biofilm (g.) was collected - depends on the flow depth **(Fig. 1)**. In combined systems the pipes are over-dimensioned to transport both wastewater and stormwater, thus the dry weather flow depth (10% of pipe diameter) is smaller than in separated systems (35% of pipe diameter) [1].

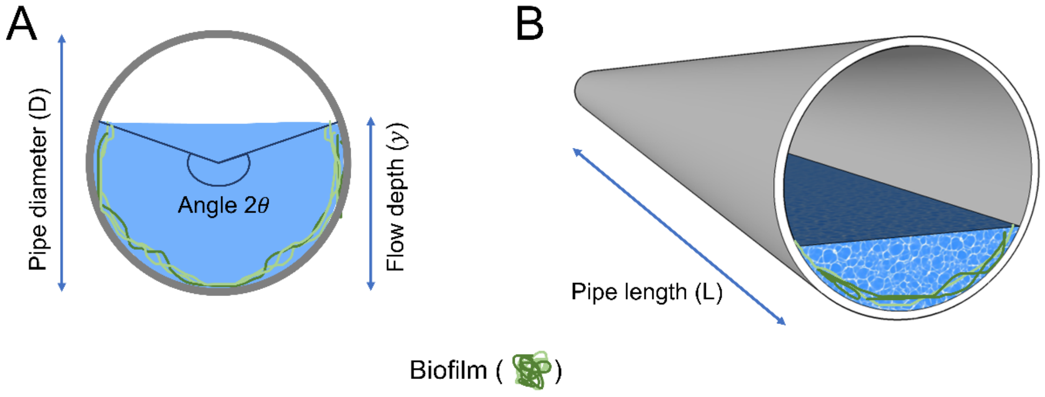

Figure 1: Illustration of the wet part of the gravity pipe where the biofilm is mostly covered by wastewater. A: Cross section of a gravity pipe. B: Illustration of the length of the pipe.

The area available for biofilm ($A_{Biofilm}$) growth in the gravity sewers of Aalborg can be calculated, where P represents the perimeter of the biofilm in a pipe cross-section and L is the pipe length.

The perimeter, P (expressed in terms of the angle θ), can be derived from the pipe diameter, D, and the flow depth, y.

$$A_{Biofilm} = PL = 2rL = DL\cos^{-1}\left(1 - \frac{2y}{D}\right) \quad (eq. 1)$$

This was done for a set of different pipe diameters representative of the sewer system in Aalborg Municipality (**Table 1**). It was assumed that the fraction of separated sewers was distributed equally among all main and branch pipes. All branch pipes were considered as gravity pipes. Sewer systems biofilm has been found to roughly contain around 10 g_{COD} m^{-2} of cell biomass [2]. Taking these distributions into account, the average biofilm mass was found to be 4.2 g cell biomass per meter sewer pipe. Scaling this to the total length of gravity sewers in Aalborg Municipality, they summed up to contain >10,000 kg of cell biomass in the biofilm.

Table 1: Details of pipe diameters in the sewer system in Aalborg Municipality. 'Pipe type' describes whether it was a main pipe or branch pipe, where branch pipes connect households and industries to main pipes. 'Fraction of each size in the system' represents the percentage of the total sewer system length comprised by each pipe diameter interval. 'Used diameter' is the diameter used for biofilm calculation. All data has been provided by Aalborg Municipality. As combined and separated systems are dimensioned differently, the biomass was calculated for each assuming that separated and combined sewers were distributed equally among all pipe types and pipe sizes.

**Pipe type**	**Pipe diameter (mm)**	**Fraction of each size in the system**	**Used diameter (mm)**	**Biofilm biomass in gravity pipes (g/m)**	
				**Combined**	**Separated**
Main	Pipelines ≤ Ø200	~50%	Ø100	0.71	1.39
Main	Ø200 < Pipelines ≤ Ø500	~40%	Ø350	2.25	4.43
Main	Ø500 < Pipelines ≤ Ø1600, majority Ø500 < Pipelines ≤ Ø800	~10%	Ø500	3.22	6.33
Branch	Ø110, Ø150 or Ø160, mostly Ø110	100%	Ø110	5.15	10.13

Supplementary references

1. Butler D et al. Urban drainage, Fourth edition. Boca Raton London New York: CRC Press, Taylor & Francis Group, 2018.
2. Hvitved-Jacobsen T, Vollertsen J, Nielsen AH. Sewer processes: Microbial and chemical process engineering of sewer networks, Second edition. CRC Press, 2013.

Supplementary tables and figures

Sewer microbiomes shape microbial community composition and dynamics of wastewater treatment plants

Marie Riisgaard-Jensen, Rodrigo Maia Valença, Miriam Peces, and Per Halkjær Nielsen

Center for Microbial Communities, Department of Chemistry and Bioscience, Aalborg University, 9220 Aalborg, Denmark

Supplementary Table 1: Overview of samples collected across sewer sampling locations and habitats. The table shows the number of unique samples collected per sampling location and sample type. Sample types include SWW (sewer wastewater), sediment (from gravity sewers), biofilm (g.) (biofilm from gravity pipes), biofilm (e.p.) (biofilm from end of pressure pipes), IWW (influent wastewater after primary settling), and AS (activated sludge from the WWTPs). The bottom row shows the total number of samples per sample type

	**SampleSite**	**IWW**	**AS**	**Biofilm (e.p.)**	**SWW**	**Biofilm (g.)**	**Sediment**	
	Aalborg East	10	10	-	-	-	-	
	Aalborg West	46	79	-	-	-	-	
	Doktorvej	-	-	3	4	-	-	
	Frejlev	-	-	-	8	7	4	
	Roden	-	-	-	5	3	4	
	Sejlflod	-	-	7	7	-	-	
	Skolesti	-	-	-	6	3	-	
	Stadionvej	-	-	-	5	2	3	
	Tvaergade	-	-	-	6	2	5	
	Visse	-	-	-	4	2	-	**Included samples:**
**Total**	—	56	89	10	45	19	16	235

Supplementary Table 2: Sewer core groups of the 50 most abundant species in activated sludge (AS) with a positive growth rate in AS. For both WWTPs, the 50 most abundant species classified as growing in AS were selected and shown with the respective sewer core group and the mean relative abundance in AS from either Aalborg West (AAW) and Aalborg East (AAE) is shown. Both the "unified core" and the habitat-based core groups are shown. Species present at ≥0.01% relative abundance in ≥80% of samples were defined as strict core species, those in ≥50% as general core species, and those in ≥20% as loose core species. "Detected" refers to species observed with any reads in >2 samples (also referred to as "detected in the sewer microbiome"). Species are sorted based on abundance in AS from AAW.

Taxonomy	Unified core	Core group per habitant			Abundance in AS (%)	
Genus; Species		Sediment	Biofilm (g.)	Biofilm (e.p.)	AAW	AAØ
Ca. Phosphoribacter;midas_s_5	loose core	detected	loose core	loose core	10.8	2.52
Ca. Microthrix parvicella	detected	detected	detected	not detected	271	1.55
Azonexus phosphoritropha	detected	not detected	detected	detected	1.08	0.3
Rhodoferax;midas_s_33	loose core	not detected	detected	loose core	1.01	0.95
Ca. Villigracilis;midas_s_471	not detected	not detected	not detected	not detected	1	1.18
Propionicimonas;midas_s_49	general core	general core	detected	general core	0.99	0.9
Ca. Microthrix subdominans	loose core	detected	detected	loose core	0.95	0.46
Ca. Amarolinea dominans	detected	not detected	detected	not detected	0.94	0.7
Ca. Brachybacter algidus	loose core	detected	loose core	detected	0.88	0.7
Nitrospira defluvii	detected	detected	detected	detected	0.75	0.72
Rhodobacter;midas_s_63	not detected	not detected	not detected	not detected	0.73	0.27
Acidovorax;midas_s_1484	loose core	detected	loose core	detected	0.69	1.19
Sphingopyxis bauzanensis	loose core	loose core	loose core	loose core	0.64	0.7
Rhodobacter;midas_s_24	detected	detected	detected	not detected	0.64	0.58
Rhodobacter;midas_s_430	general core	general core	general core	general core	0.6	0.47
midas_g_179;midas_s_179	loose core	detected	detected	loose core	0.58	0.39
midas_g_171;midas_s_171	detected	not detected	detected	not detected	0.53	0.18
Azonexus phosphorivorans	general core	loose core	loose core	general core	0.51	1.11
Ahniella;midas_s_116	detected	detected	detected	not detected	0.5	0.77
Tabrizicola aquatica	strict core	detected	strict core	general core	0.5	0.15
Ca. Promineofilum glycogenico	detected	detected	detected	detected	0.48	0.29
midas_g_20;midas_s_20	not detected	not detected	not detected	not detected	0.47	0.21
midas_g_70;midas_s_70	loose core	detected	detected	loose core	0.46	0.1
Leptothrix;midas_s_81	not detected	not detected	not detected	not detected	0.44	0.24
Nitrotoga;midas_s_181	detected	not detected	detected	not detected	0.4	0.82
Ca. Amarobacter glycogenicus	not detected	not detected	not detected	not detected	0.38	0.18
Terrimonas;midas_s_743	detected	not detected	detected	not detected	0.36	0.18

Thermomonas;midas_s_203	loose core	detected	loose core	loose core	0.32	0.15
AAP99;midas_s_215	detected	detected	not detected	not detected	0.3	0.07
Lautropia;midas_s_135	not detected	not detected	not detected	not detected	0.29	0.25
midas_g_385;midas_s_385	detected	not detected	detected	detected	0.26	0.31
midas_g_422;midas_s_742	detected	not detected	detected	not detected	0.26	0.2
Haliscomenobacter hydrossis	detected	not detected	detected	not detected	0.25	0.16
midas_g_321;midas_s_321	not detected	not detected	not detected	not detected	0.24	0.1
Nannocystis;midas_s_1119	detected	not detected	detected	not detected	0.24	0.2
midas_g_2010;midas_s_2010	not detected	not detected	not detected	not detected	0.23	0.18
midas_g_973;midas_s_973	detected	detected	detected	detected	0.23	0.03
midas_g_59;midas_s_59	not detected	not detected	not detected	not detected	0.22	0.46
Nitrosomonas;midas_s_717	detected	not detected	not detected	detected	0.22	0.19
Falsirhodobacter;midas_s_1464	detected	detected	detected	not detected	0.21	0.07
JGI 0001001-H03;midas_s_170	detected	not detected	not detected	detected	0.21	0.15
Ca. Accumulibacter phosphatis	loose core	not detected	detected	loose core	0.2	0.63
midas_g_33;midas_s_50	detected	detected	detected	not detected	0.2	0.04
midas_g_155;midas_s_278	loose core	detected	loose core	loose core	0.19	0.46
midas_g_1341;midas_s_3347	detected	detected	detected	not detected	0.19	0.22
Novosphingobium;midas_s_1084	detected	not detected	detected	detected	0.19	0.07
Sphingopyxis;midas_s_983	detected	not detected	detected	not detected	0.19	0.13
Ca. Sarcinithrix;midas_s_425	detected	detected	detected	not detected	0.19	0.08
Mesorhizobium;midas_s_967	loose core	detected	loose core	loose core	0.18	0.08
midas_g_5535;midas_s_5535	not detected	not detected	not detected	not detected	0.18	0.13
midas_g_2835;midas_s_2835	detected	not detected	detected	not detected	0.15	0.24
midas_g_428;midas_s_428	detected	not detected	not detected	detected	0.13	0.2
Sulfuritalea hydrogenivorans	not detected	not detected	not detected	not detected	0.13	0.18
Rhodoferax;midas_s_320	general core	detected	general core	general core	0.12	0.45
midas_g_111;midas_s_111	not detected	not detected	not detected	not detected	0.12	0.18
Ca. Saccharimonas aalborgensis	not detected	not detected	not detected	not detected	0.11	0.22
midas_g_81;midas_s_202	loose core	detected	loose core	loose core	0.11	0.18
Defluviimonas;midas_s_1678	general core	detected	loose core	general core	0.1	0.24
Uliginosibacterium;midas_s_53	detected	not detected	detected	not detected	0.08	0.26
Nitrosomonas;midas_s_139	detected	not detected	detected	not detected	0.07	0.67
midas_g_57;midas_s_57	detected	detected	detected	not detected	0.05	0.45
Denitratisoma;midas_s_5897	not detected	not detected	not detected	not detected	0.02	0.19
midas_g_6877;midas_s_7259	detected	detected	detected	detected	0	0.27
midas_g_93;midas_s_93	not detected	not detected	not detected	not detected	0	0.25
Methylorosula;midas_s_163	loose core	detected	not detected	loose core	0	0.23

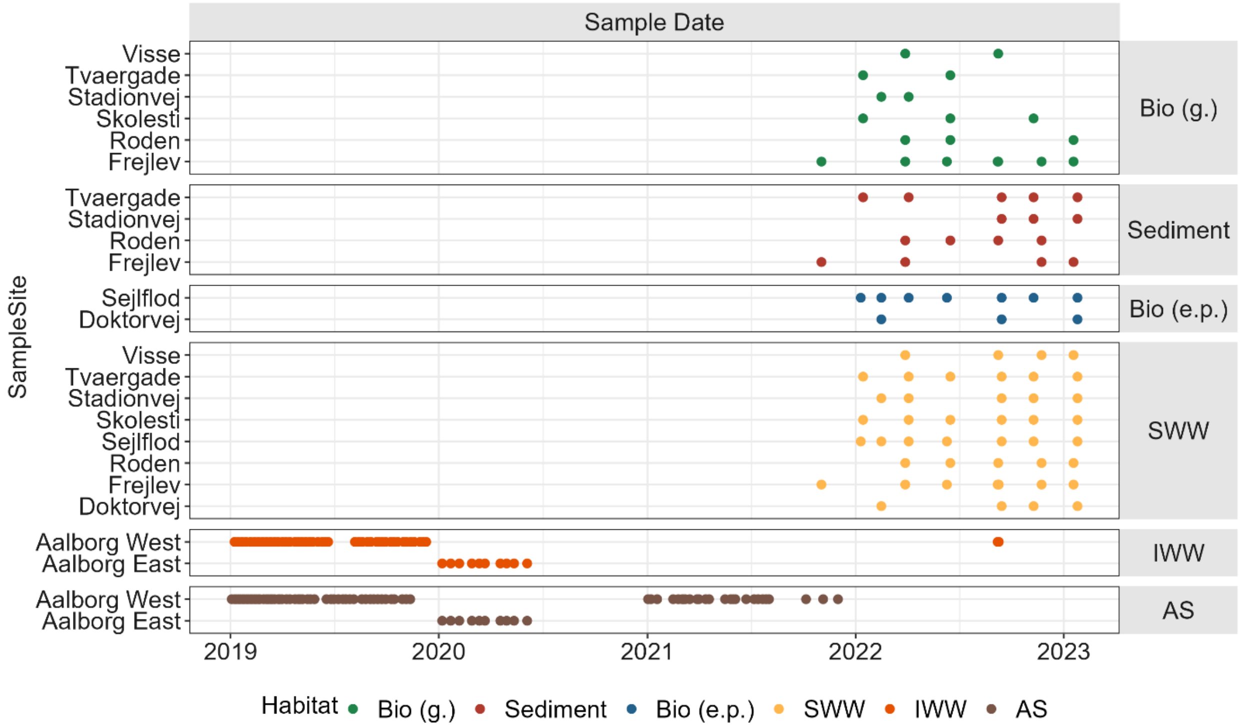

Figure S1: Sampling timeline. Every dot represents a sample point. Biofilm is abbreviated as Bio.

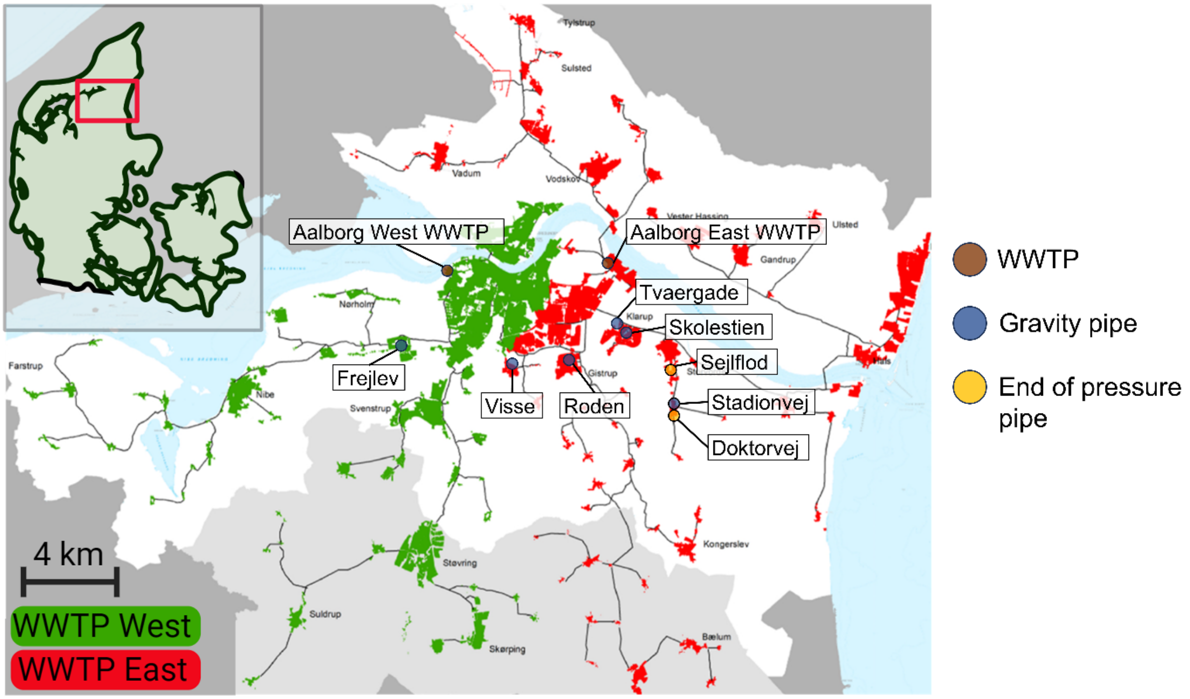

Figure S2: Sampling locations. Map of the catchment area in Aalborg Municipality, showing the catchments of Aalborg East and Aalborg West WWTPs in red and green, respectively. Each dot represents a sampling location, with the dot color indicating the type of sewer. Sampling locations are labeled with their corresponding names.

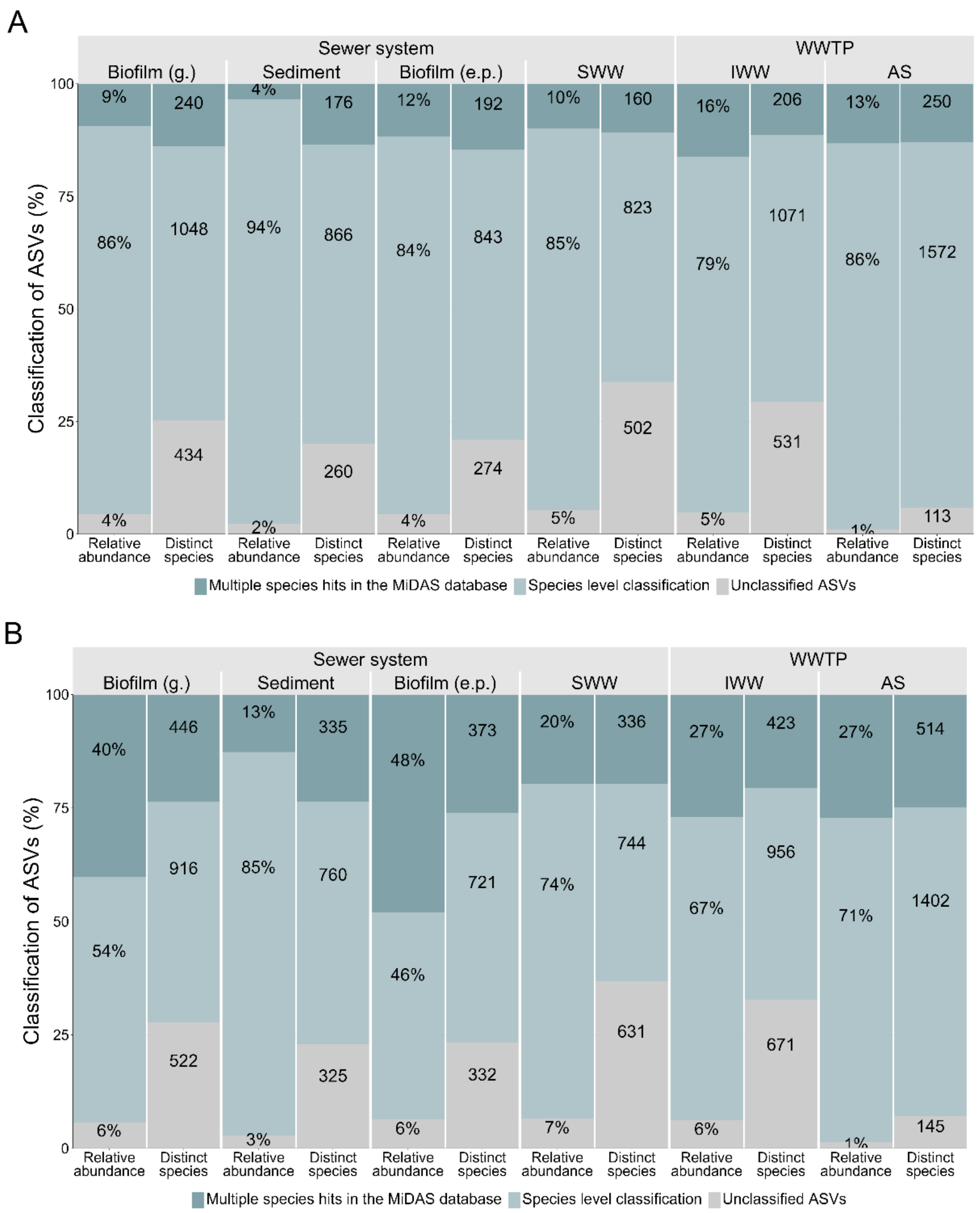

Figure S3: Species-level classification of ASVs using the MiDAS database. The ASVs have been mapped to the database using (A) the usearch *-sintax 0.6* command and (B) the usearch -*sintax 0.8* command. Subsequently, the unclassified ASV were mapped to the database using usearch with an identity cut-off of 98.7% to investigate if the ASV were unclassified due to no database hit (Unclassified ASVs) or due to the inability to distinguish between two or more species in the database (Multiple species hits in the MiDAS database v.5.3). "Relative abundance" is the cumulative abundance of species/ASVs within each group while "Distinct species" are the number of distinct species or unclassified ASVs (transformed to %) with the absolute number shown for each group.

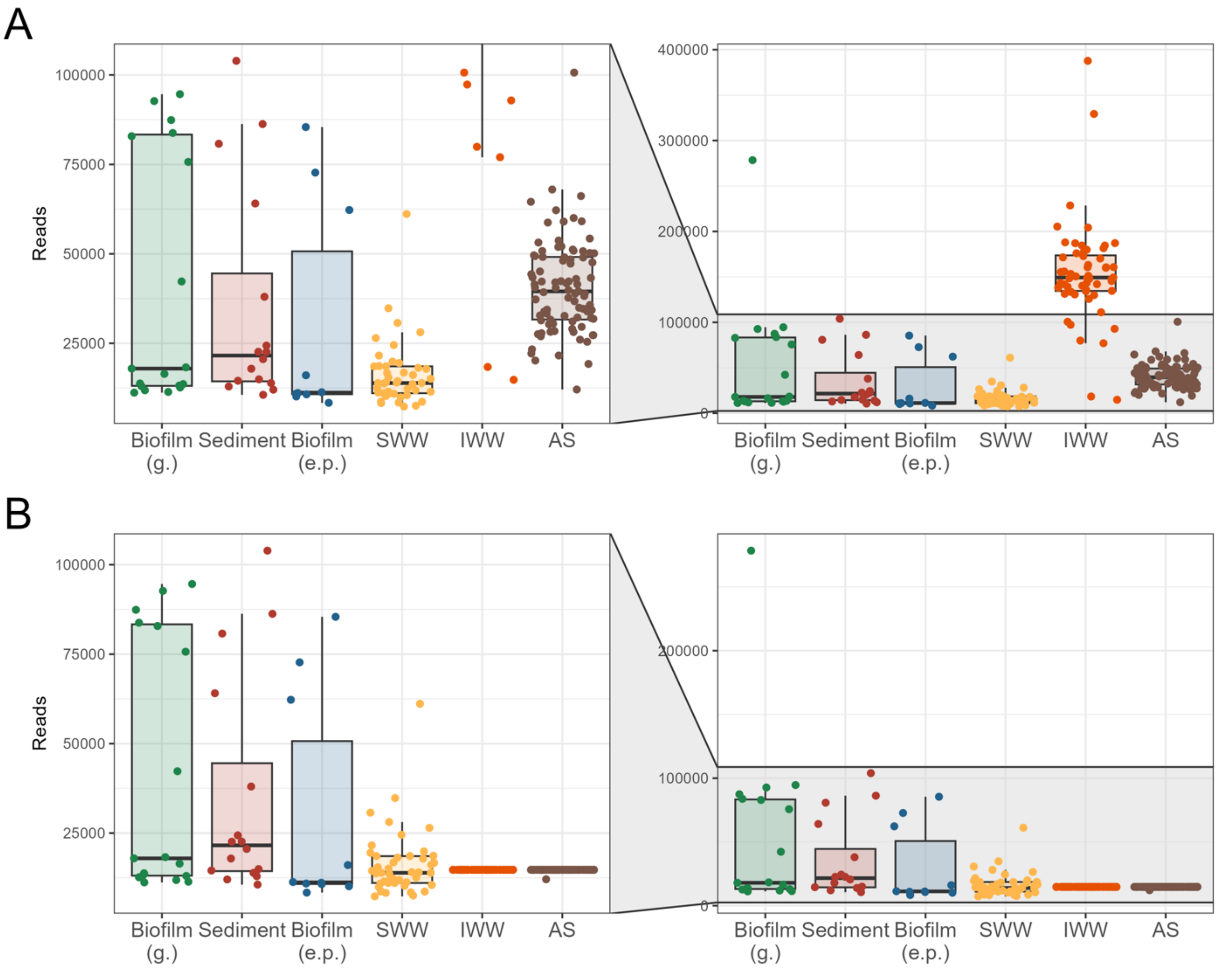

Figure S4: Number of sequencing reads per habitat. A: Reads before rarefaction of IWW and AS. B: Reads after rarefaction of IWW and AS.

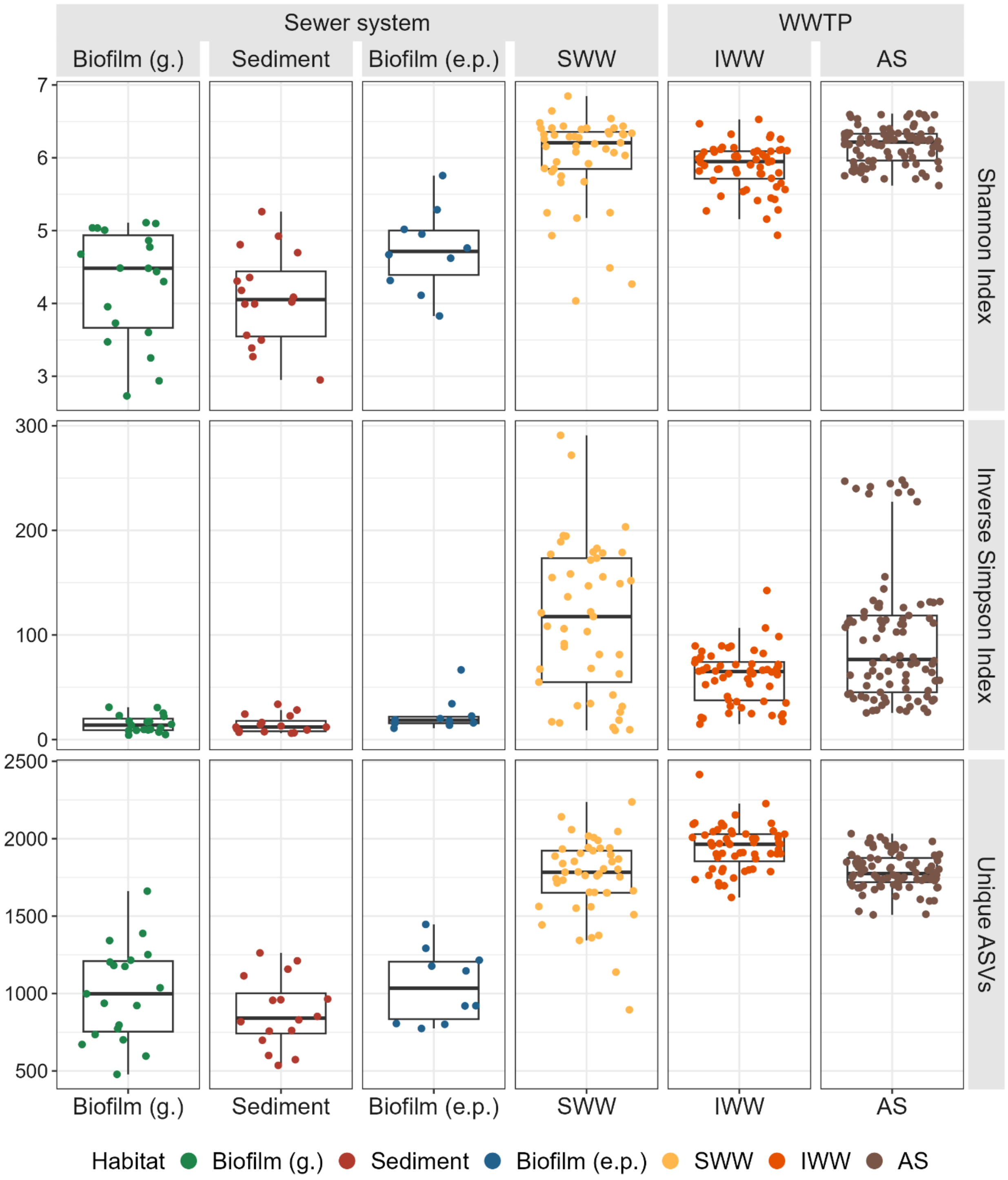

Figure S5: Alpha-diversity measures. Shannon Index, Inverse Simpson Index, and the number of unique ASVs are shown for each habitat. All samples are rarefied to 7322 reads.

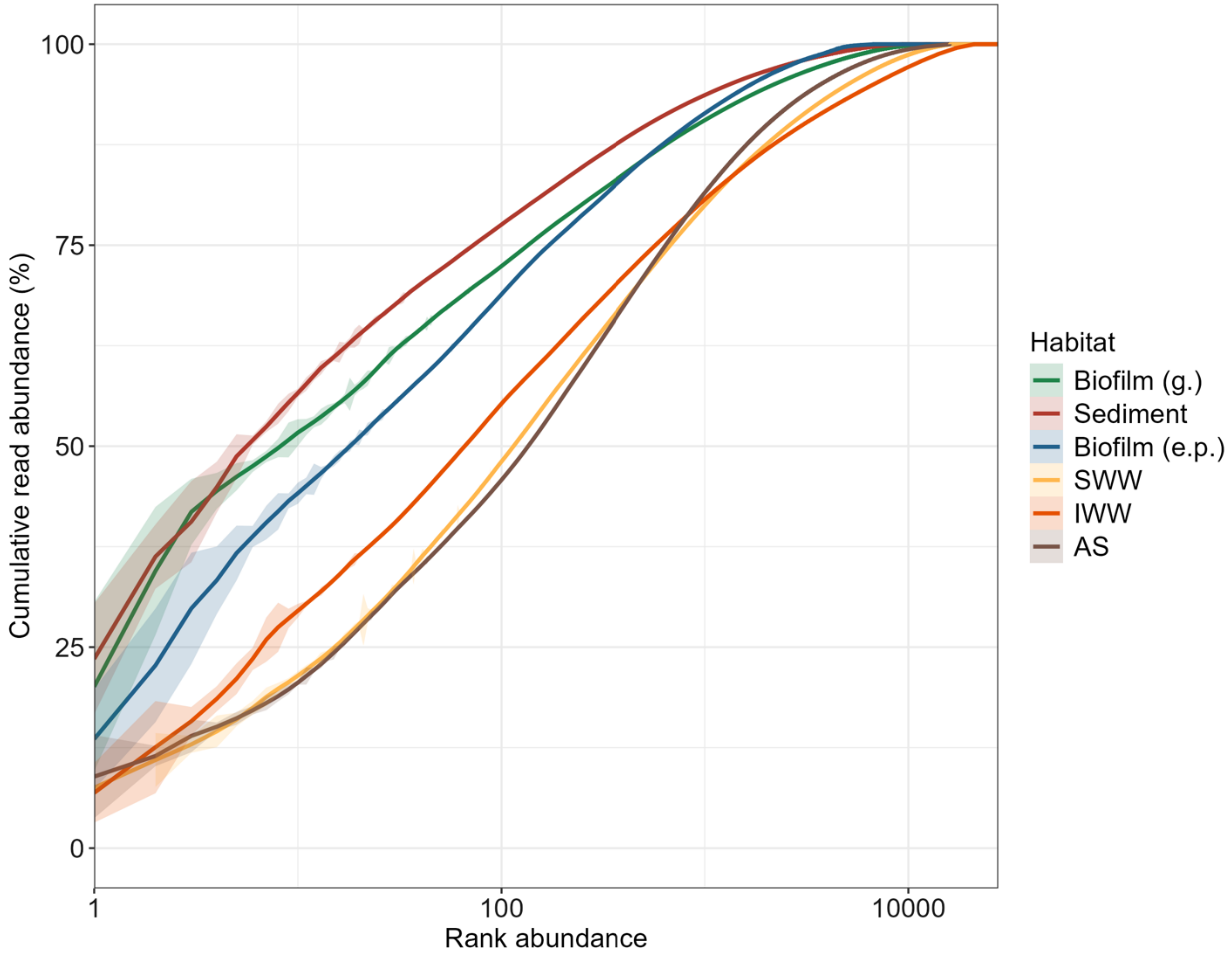

Figure S6: Rank abundance versus cumulative read abundance. Each line represents the mean of a habitat with standard deviation intervals from the mean shown as transparent shading.

	Sewer system				WWTP		
	Biofilm (g.)	Sediment	Biofilm (e.p.)	SWW	IWW	AS	
Trichococcus	25.24	41.80	12.47	12.92	12.40	2.14	All
Acidovorax	22.86	4.70	16.47	1.92	3.78	1.86	All
Thiothrix	0.48	0.11	18.48	0.08	0.16	0.13	Sewer env.
Simplicispira	6.55	1.63	2.48	0.58	0.69	0.20	Sewer env.
Proteiniclasticum	1.78	4.66	1.06	0.43	0.36	0.10	Sewer env.
Thauera	1.17	5.22	0.52	0.22	0.74	0.44	Sewer env.
Christensenellaceae_R-7_group	1.33	2.18	1.31	0.84	0.61	0.23	Sewer env.
Desulfobulbus	0.52	3.91	0.17	0.06	0.03	0.02	Sewer env.
Comamonas	1.74	0.23	1.03	0.34	1.22	0.14	Sewer env.
Leptotrichia	0.97	0.44	1.13	0.48	0.84	0.11	Sewer env.
Novosphingobium	0.22	0.02	2.50	0.03	0.03	0.99	Sewer env.
Pseudorhodobacter	0.61	0.14	2.47	0.10	0.21	0.18	Sewer env.
Acetobacterium	1.16	1.42	0.23	0.22	0.14	0.03	Sewer env.
Brooklawnia	0.13	2.16	0.17	0.07	0.03	0.04	Sewer env.
Aestuariimicrobium	0.27	1.66	0.16	0.11	0.04	0.07	Sewer env.
Polaromonas	1.24	0.06	0.49	0.12	0.10	0.04	Sewer env.
Anaerolineaceae; midas_g_467	0.20	1.63	0.16	0.02		0.02	Sewer env.
Blautia	0.19	0.12	0.14	12.51	5.83	0.20	Wastewater
Streptococcus	0.25	0.17	0.12	2.00	9.69	0.40	Wastewater
Arcobacter	0.73	1.05	0.23	1.54	8.57	0.21	Wastewater
Faecalibacterium	0.12	0.06	0.10	4.91	3.82	0.09	Wastewater
Subdoligranulum	0.12	0.09	0.12	4.05	2.08	0.23	Wastewater
Agathobacter	0.05	0.02	0.03	3.82	1.97	0.09	Wastewater
Ruminococcus	0.11	0.08	0.07	1.90	1.37	0.15	Wastewater
Dorea	0.07	0.03	0.05	2.43	1.00	0.06	Wastewater
Fusicatenibacter	0.10	0.04	0.07	1.97	1.16	0.09	Wastewater
Pseudarcobacter	0.28	0.21	0.09	0.44	1.84	0.10	Wastewater
Brachymonas	0.19	0.06	0.06	0.06	1.73	0.06	Wastewater
Rhodoferax	2.45	0.29	2.51	0.38	0.44	4.78	Sewer env. + AS
Azonexus	0.29	0.11	1.49	0.08	0.12	2.15	Sewer env. + AS
Ca. Phosphoribacter			0.03	0.02	0.01	9.93	AS
Rhodobacter	0.19	0.08	0.32	0.06	0.10	3.02	AS
Ca. Microthrix			0.01		0.01	3.48	AS
Ca. Villigracilis						1.32	AS
Ca. Opimibacter						1.05	AS
Ca. Amarolinea						1.01	AS
Remaining abundance	32.05	26.97	33.84	45.21	39.13	65.01	
Remaining genera	963	682	622	749	798	904	

Relative abundance (%): >10, 5-10, 1-5, 0.1-1, 0.01-0.1, 0-0.01, 0

Figure S7: Top 10 most abundant bacterial genera in all habitats. The top 10 genera were selected separately for each habitat, resulting in a total of 36 unique genera. Facet labels indicate in which habitat the genera are part of the top 10: "All": top 10 in all habitats; "Sewer env.": top 10 in any biofilm (g.), biofilm (e.p.), and sediment; "Wastewater": IWW and/or SWW; "Sewer env. + AS": in either biofilm (g.), biofilm (e.p.), sediment, and in AS; "AS": Activated sludge. Abundances are shown with two decimals if ≥0.01%. If no reads were found the abundance is 0%. "Remaining abundance" and "Remaining genera" refers to the cumulative abundance or number of genera outside of the top 10, respectively. Only classified genera are shown. For MiDAS placeholder names, the corresponding family name is provided.

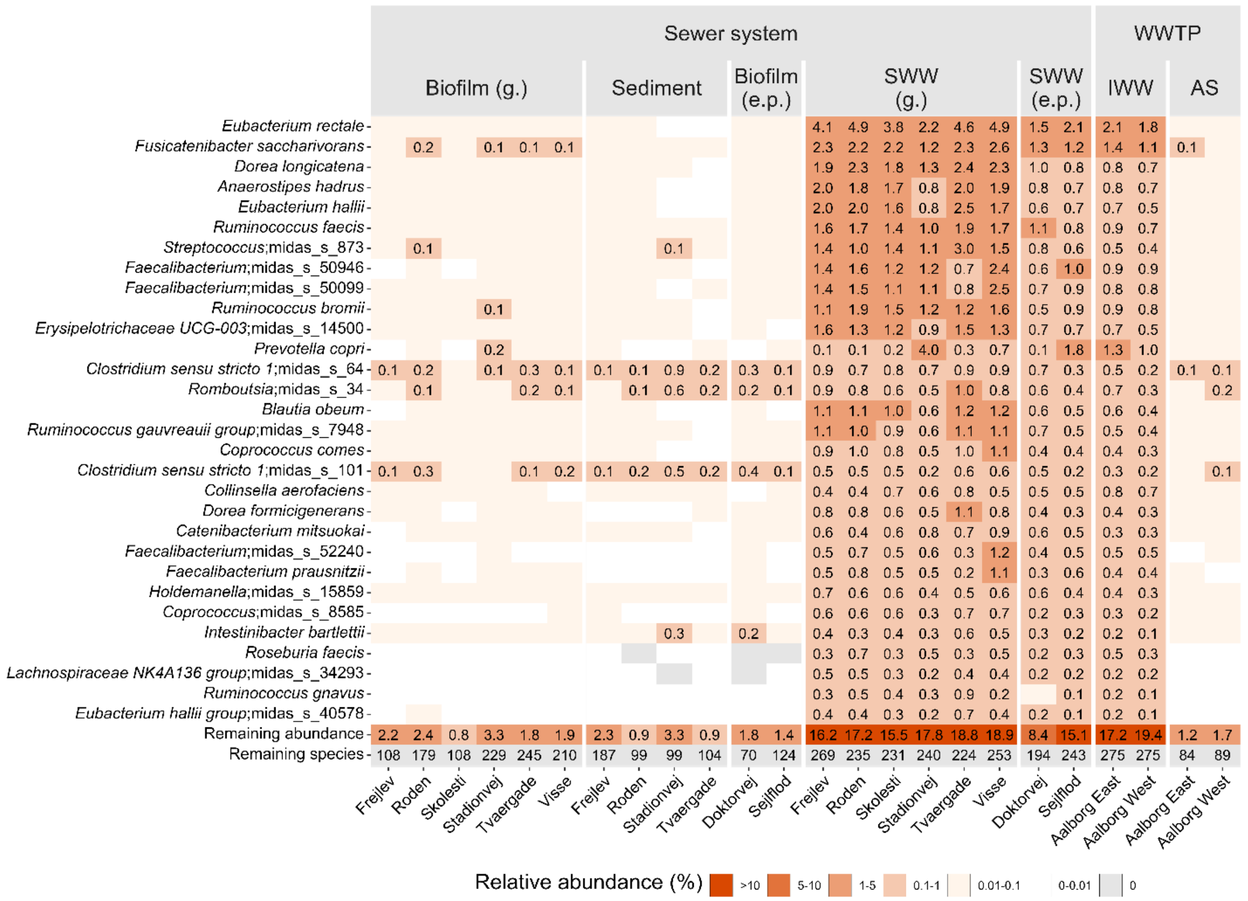

Figure S8: Top 30 most abundant gut bacterial species in SWW. Abundances are shown with one decimal if ≥0.1%. If no reads were found the abundance is 0%. "Remaining abundance" and "Remaining species" refer to the cumulative abundance or number of gut species outside of the top 30, respectively. Only classified genera are shown. For MiDAS placeholder names, the corresponding family name is provided.

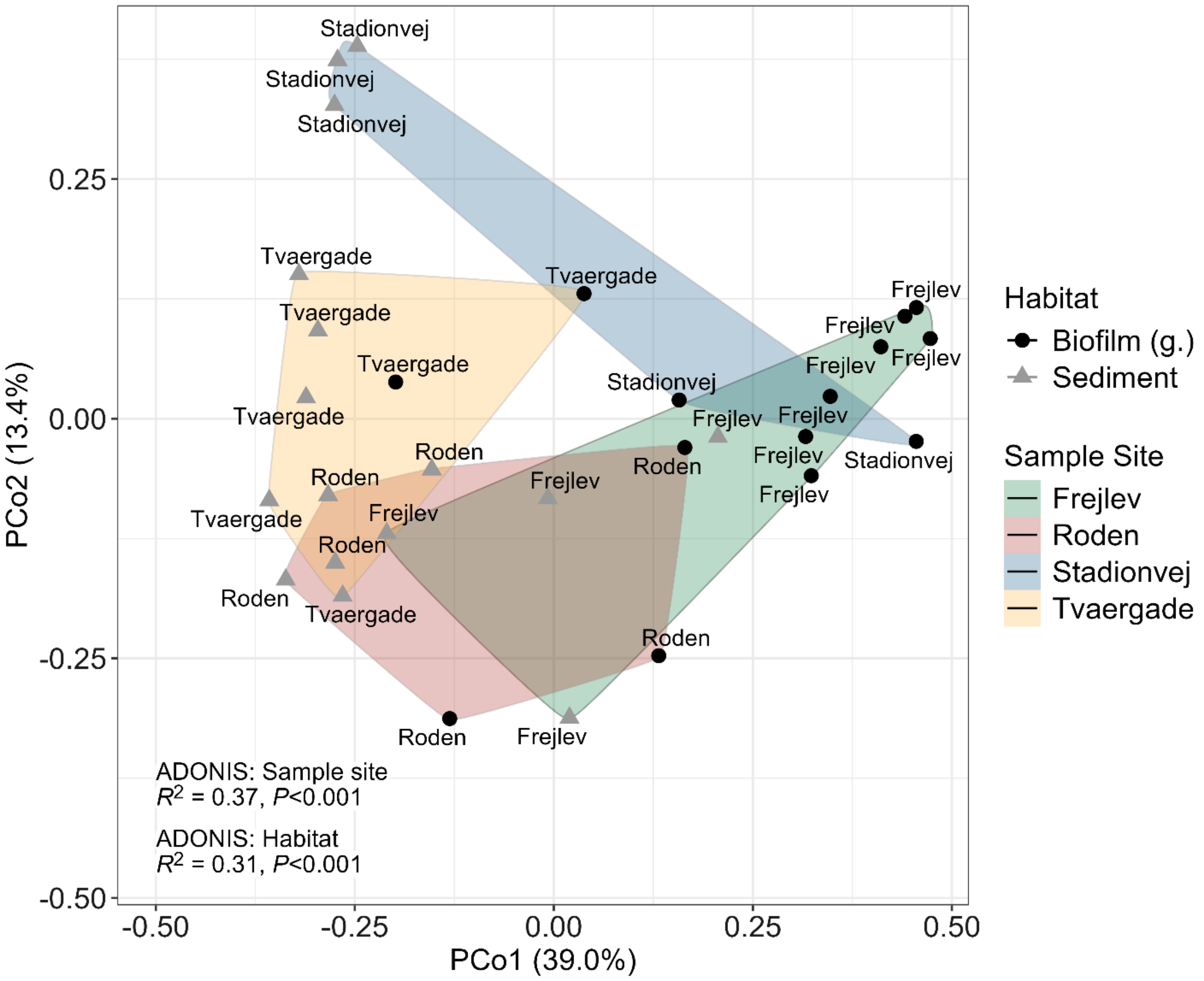

Figure S9: Beta diversity of biofilm (g.) and sediments across the sample sites where both types were collected. Samples are grouped and colored based on the sample site and the points are colored and shaped by habitat (biofilm (g.) or sediment). The PCoA was made at the species level using the Bray-Curtis distance matrix (relative abundance > 0.01%). An ADONIS analysis showed habitat and sample site to explain 31% and 37% (p<0.001) of the microbial community variation, respectively.

Relative abundance (%) — Sewer system

Species	Frejlev Biofilm (g.)	Frejlev Sediment	Roden Biofilm (g.)	Roden Sediment	Stadionvej Biofilm (g.)	Stadionvej Sediment	Tvaergade Biofilm (g.)	Tvaergade Sediment
Trichococcus;midas_s_4	18.98	42.44	46.31	50.07	20.01	25.52	30.78	44.40
Acidovorax;midas_s_2077	28.84	11.75	10.99	3.13	14.50	0.30	9.70	1.88
Thauera;midas_s_256	0.07	2.33	0.34	5.64	1.43	5.32	2.01	4.31
Christensenellaceae R-7 group;midas_s_785	0.96	0.65	0.51	1.64	0.11	0.94	1.97	1.82
Desulfobulbus;midas_s_1262	0.25	1.41	0.08	3.10	0.14	3.27	2.82	6.70
Simplicispira psychrophila	6.08	2.35	3.38	0.51	3.35	0.01	0.49	0.20
Proteiniclasticum;midas_s_1025	0.39	0.72	0.85	2.61	0.06	1.72	3.05	3.69
Simplicispira;midas_s_2606	2.53	2.44	1.72	0.44	0.89		0.16	0.18
Proteiniclasticum;midas_s_140	0.24	0.73	0.53	1.36	0.14	1.01	1.64	1.68
Thauera terpenica	0.47	1.24	0.53	0.53	1.19	0.51	0.93	0.62
Arcobacter;midas_s_1292	0.02	1.09	0.47	1.30	1.36	0.03	0.77	0.43
Aestuariimicrobium;midas_s_338	0.16	0.30	0.18	1.12	0.03	3.37	0.82	2.05
Paludibacter;midas_s_2705	0.04	0.24	0.12	1.11	0.49	1.65	2.79	1.52
Brooklawnia;midas_s_193	0.02	0.41	0.03	1.25	0.14	3.73	0.74	1.48
Rhodoferax;midas_s_10145	0.36	0.18	0.69	0.03	2.22		0.03	
Leptotrichia;midas_s_2907	0.25	0.79	0.60	0.36	1.05	0.26	0.98	0.34
midas_g_249;midas_s_64864	0.15	0.21	0.10	0.46	0.07	1.46	0.94	0.78
Enterococcus aquimarinus	0.44	0.76	0.39	0.28	1.25	0.08	0.40	0.13
Rhodoferax;midas_s_1744	0.26	0.45	0.99	0.05	0.41	0.01	0.26	0.08
Acetobacterium wieringae	0.54	0.44	0.34	0.55	0.07	0.20	0.70	0.81
Actinomyces;midas_s_4035	0.13	0.72	0.10	0.16	0.29	1.40	0.18	0.29
Lactivibrio;midas_s_3664	0.02	0.11		0.29	0.03	0.49	0.95	0.97
midas_g_49;midas_s_572		0.12	0.04	0.81		1.13	0.21	0.28
Pseudarcobacter;midas_s_1440		0.10	0.19	0.41	1.04	0.03	0.37	0.13
midas_g_467;midas_s_4730	0.06	0.10	0.02	0.34		1.05	0.58	0.96
Christensenellaceae R-7 group;midas_s_3317	0.17	0.15	0.10	0.41	0.04	0.73	0.35	0.39
Clostridium sensu stricto 1;midas_s_64	0.11	0.14	0.22	0.12	0.10	0.91	0.27	0.25
Flavobacterium;midas_s_6887	0.23	0.60	0.24	0.05	0.67	0.03	0.21	0.06
midas_g_164;midas_s_164		0.05		0.24	0.01	1.14	0.08	0.23
Brooklawnia;midas_s_845		0.08	0.05	0.85		0.41	0.07	0.30
Tessaracoccus;midas_s_413	0.07	0.18	0.18	0.50	0.02	0.59	0.05	0.11
midas_g_467;midas_s_1532		0.03		0.08	0.01	1.14	0.21	0.23
Simplicispira piscis	0.83	0.07	0.18		0.52	0.02	0.05	0.01
Rivicola;midas_s_6762		0.03	0.04	0.18	1.40			
Ca. Competibacter;midas_s_5409	0.22		0.03	0.02		0.02	1.19	0.13
Aquabacterium;midas_s_333	0.50	0.21	0.12	0.04	0.57		0.10	0.01
Lactococcus raffinolactis	0.10	0.13	0.53	0.26	0.10	0.04	0.16	0.06
Zoogloea;midas_s_1080	0.01	0.06	0.12	0.03	1.09		0.03	0.01
Polaromonas glacialis	0.04	0.02	0.50		0.44			
Cereibacter;midas_s_3965	0.18	0.17	0.29	0.02	0.24		0.03	0.02
Giesbergeria;midas_s_27419	0.01	0.26	0.59		0.04			
midas_g_2342;midas_s_2342	0.10	0.18	0.34	0.03	0.10		0.07	0.05
Brooklawnia;midas_s_83590		0.03	0.02	0.40				0.02
Variovorax;midas_s_115917	0.28							
Remaining abundance	41.68	29.42	34.03	19.58	44.03	42.07	32.67	22.31
Remaining species	2042	2330	2153	1182	2717	1200	3601	1439

Relative abundance (%): >10; 5-10; 1-5; 0.1-1; 0.01-0.1; 0-0.01; 0

Figure S10: Top 30 most abundant bacterial species in each of the sample sites where both sediment and biofilm (g) were collected. The top 30 species were selected separately for each sample site, resulting in a total of 44 unique species. Abundances are shown with two decimals if ≥0.01%. If no reads were found the abundance is 0%. "Remaining abundance" and "Remaining species" refers to the cumulative abundance or number of species outside of the top 30, respectively. Only classified species are included, with genus names provided for species classified with MiDAS placeholder names. To retrieve the 30 most abundant species, each site was subsampled to include the same number of biofilm (g.) and sediment samples.

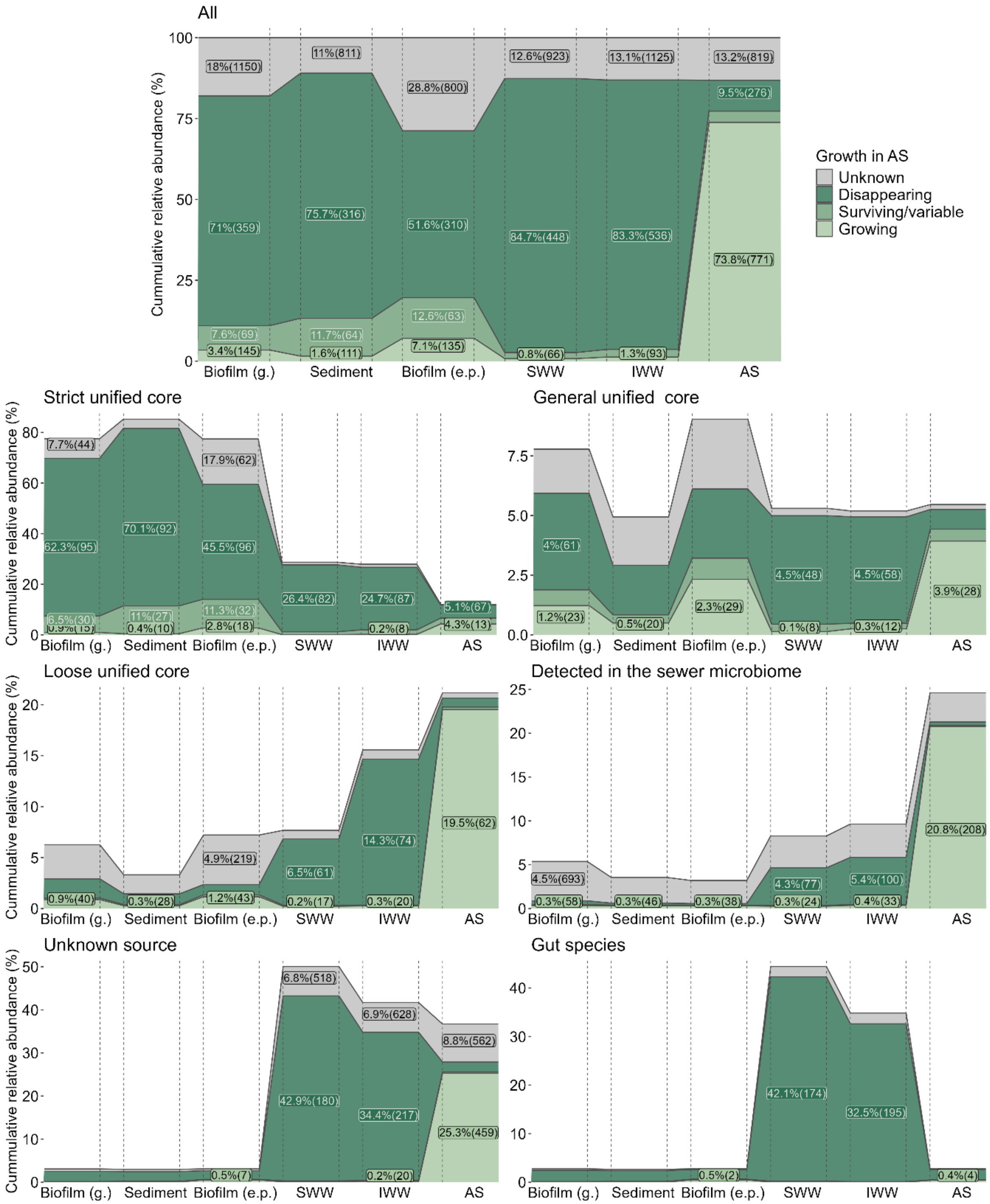

Figure S11: Cumulative abundance of bacterial species based on their growth fate and source in AS, shown either merged ("All") or divided according to the sewer unified core groups. "All" represents the full dataset, while the smaller plots below show subsets based on the individual sewer core groups. Bars represent the average relative abundance for each group. If the cumulative relative abundance exceeds 4%, or is ≥0.1% for species growing in AS, the value is displayed along with the number of species in parentheses.

Species	Sewer system: Biofilm (g.)	Sewer system: Sediment	Sewer system: Biofilm (e.p.)	Sewer system: SWW	WWTP: IWW	WWTP: AS	Facet
Trichococcus;midas_s_4	25.24	41.79	12.46	12.91	12.40	2.14	All
Acidovorax;midas_s_2077	20.13	4.36	13.60	1.62	2.50	0.64	Sewer env.+Wastewater
Thiothrix lacustris	0.40	0.10	9.53	0.05	0.05	0.04	Sewer env.
Simplicispira psychrophila	4.43	0.78	1.27	0.28	0.56	0.11	Sewer env.
Thauera;midas_s_256	0.52	4.34	0.11	0.06	0.10	0.10	Sewer env.
Desulfobulbus;midas_s_1262	0.50	3.83	0.13	0.05	0.02	0.02	Sewer env.
Leptotrichia;midas_s_2907	0.97	0.44	1.11	0.48	0.82	0.11	Sewer env.
Proteiniclasticum;midas_s_1025	0.83	2.31	0.48	0.08	0.04	0.03	Sewer env.
Thiothrix;midas_s_1351	0.01		3.57		0.03	0.04	Sewer env.
Simplicispira;midas_s_2606	1.45	0.78	0.38	0.26	0.06	0.05	Sewer env.
Christensenellaceae R-7 group;midas_s_785	0.91	1.32	0.52	0.11	0.06	0.05	Sewer env.
Comamonas denitrificans	1.17	0.14	0.24	0.21	0.94	0.08	Sewer env.
Thiothrix unzii	0.03		2.21	0.02	0.04	0.02	Sewer env.
Proteiniclasticum;midas_s_140	0.56	1.23	0.25	0.08	0.08	0.04	Sewer env.
Paludibacter;midas_s_2705	0.41	1.12	0.54	0.01	0.02		Sewer env.
Aestuariimicrobium;midas_s_338	0.25	1.63	0.07	0.07	0.03	0.02	Sewer env.
Brooklawnia;midas_s_193	0.11	1.58	0.14	0.05	0.02	0.03	Sewer env.
Rhodoferax;midas_s_10145	0.70	0.05	0.96	0.06	0.07	0.03	Sewer env.
Thiothrix;midas_s_525	0.03		1.64		0.03	0.03	Sewer env.
Thiothrix;midas_s_37971			0.99				Sewer env.
Polaromonas;midas_s_4525	0.78	0.04	0.03	0.06	0.05	0.02	Sewer env.
Streptococcus parasuis				0.03	5.82	0.15	Wastewater
Eubacterium rectale	0.04	0.02	0.03	3.52	1.81	0.08	Wastewater
Subdoligranulum;midas_s_348	0.10	0.06	0.10	3.17	1.43	0.18	Wastewater
Enterococcus aquimarinus	0.52	0.31	0.45	1.40	0.75	0.08	Wastewater
Fusicatenibacter saccharivorans	0.10	0.04	0.07	1.93	1.14	0.09	Wastewater
Dorea longicatena	0.05	0.02	0.03	1.71	0.69	0.04	Wastewater
Arcobacter;midas_s_2255	0.08	0.06	0.02	0.29	1.89	0.02	Wastewater
Anaerostipes hadrus	0.06	0.02	0.05	1.48	0.69	0.05	Wastewater
Ruminococcus faecis	0.05	0.02	0.05	1.40	0.70	0.05	Wastewater
Eubacterium hallii	0.02	0.01	0.03	1.53	0.54	0.03	Wastewater
Prevotella copri	0.04	0.01	0.01	0.90	1.02	0.03	Wastewater
Brachymonas denitrificans					1.57	0.06	Wastewater
Pannonibacter phragmitetus					1.07	0.04	Wastewater
Ca. Phosphoribacter;midas_s_5			0.03	0.02	0.01	9.87	AS
Ca. Microthrix parvicella						2.58	AS
Propionicimonas;midas_s_49		0.10	0.03		0.01	0.98	AS
Ca. Opimibacter;midas_s_17						1.05	AS
Ca. Villigracilis;midas_s_471						1.02	AS
Rhodoferax;midas_s_33			0.02			1.00	AS
Azonexus phosphoritropha						0.99	AS
Ca. Microthrix subdominans			0.01			0.89	AS
Ca. Amarolinea dominans						0.91	AS
Remaining abundance	44.8	35.4	49.8	66.9	63.1	76.4	
Remaining species	2408	1681	1498	1650	1793	1905	

Relative abundance (%): >10, 5-10, 1-5, 0.1-1, 0.01-0.1, 0-0.01, 0

Figure S12: Top 10 most abundant bacterial species in each habitat. The top 10 species were selected separately for each habitat, resulting in a total of 43 unique species. The facets (y-axis) indicate in which habitats the species are part of the top 10: "All": All habitats; "Sewer env.": Any of biofilm (g.), biofilm (e.p.), and sediment; "Wastewater": IWW and/or SWW; "AS": AS. Abundances are shown with two decimals if ≥0.01%. If no reads were found the abundance is 0%. "Remaining abundance" and "Remaining species" refers to the cumulative abundance or number of species outside of the top 10, respectively. Only classified species are included, with genus names provided for species classified with MiDAS placeholder names.

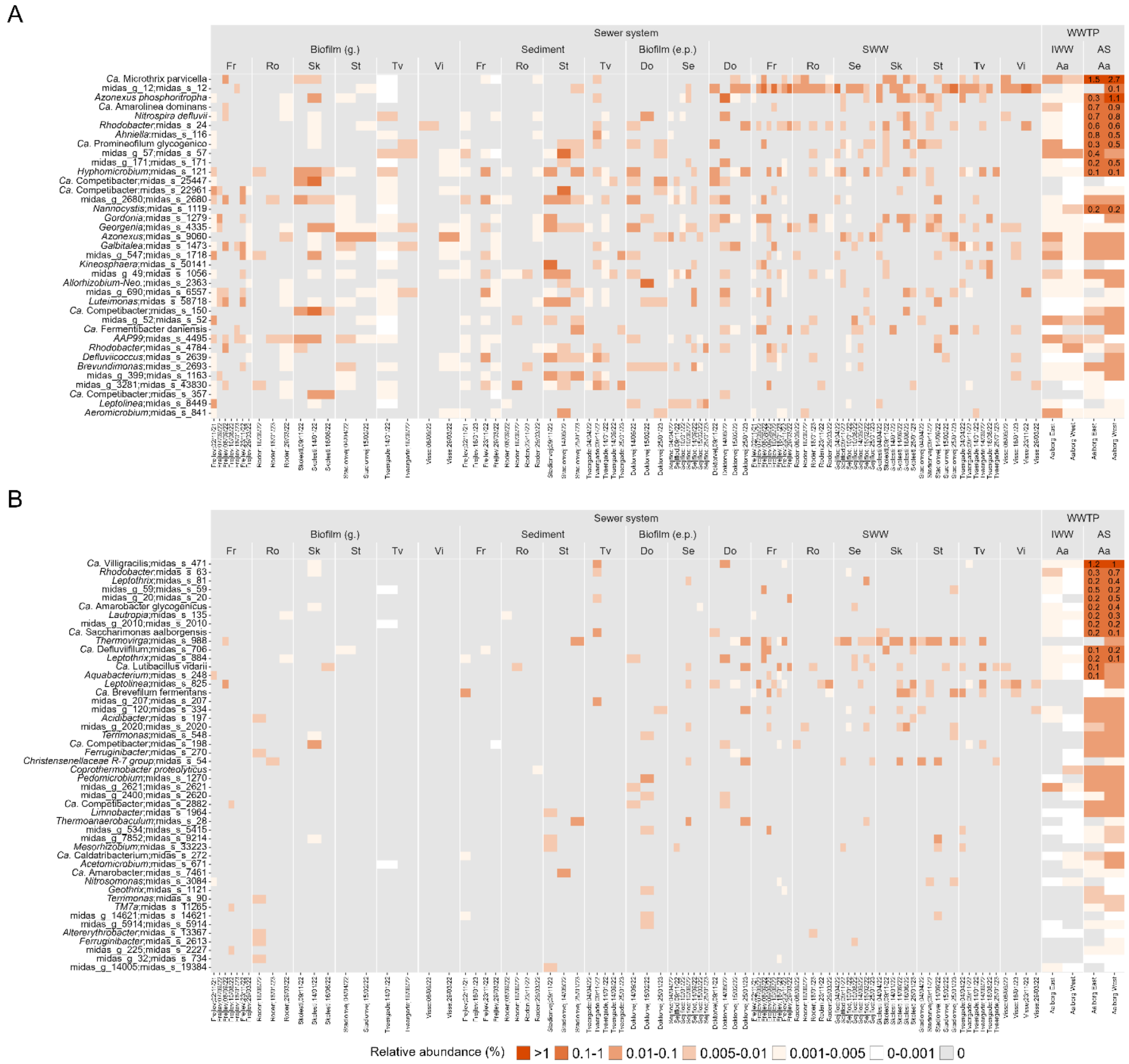

Figure S13: Top 8 most abundant bacterial species in each habitat that were determined to be growing in AS and were either (A) detected or (B) not detected in the sewer microbiome. The top 8 species were selected separately for each habitat, resulting in 37 unique species in (A) and 48 in (B). In samples from WWTPs, abundances are shown with labels if >0.01%; if no reads were detected, the abundance is shown as 0%. In the facet labels, sample sites are abbreviated using the first two letters of each site name.

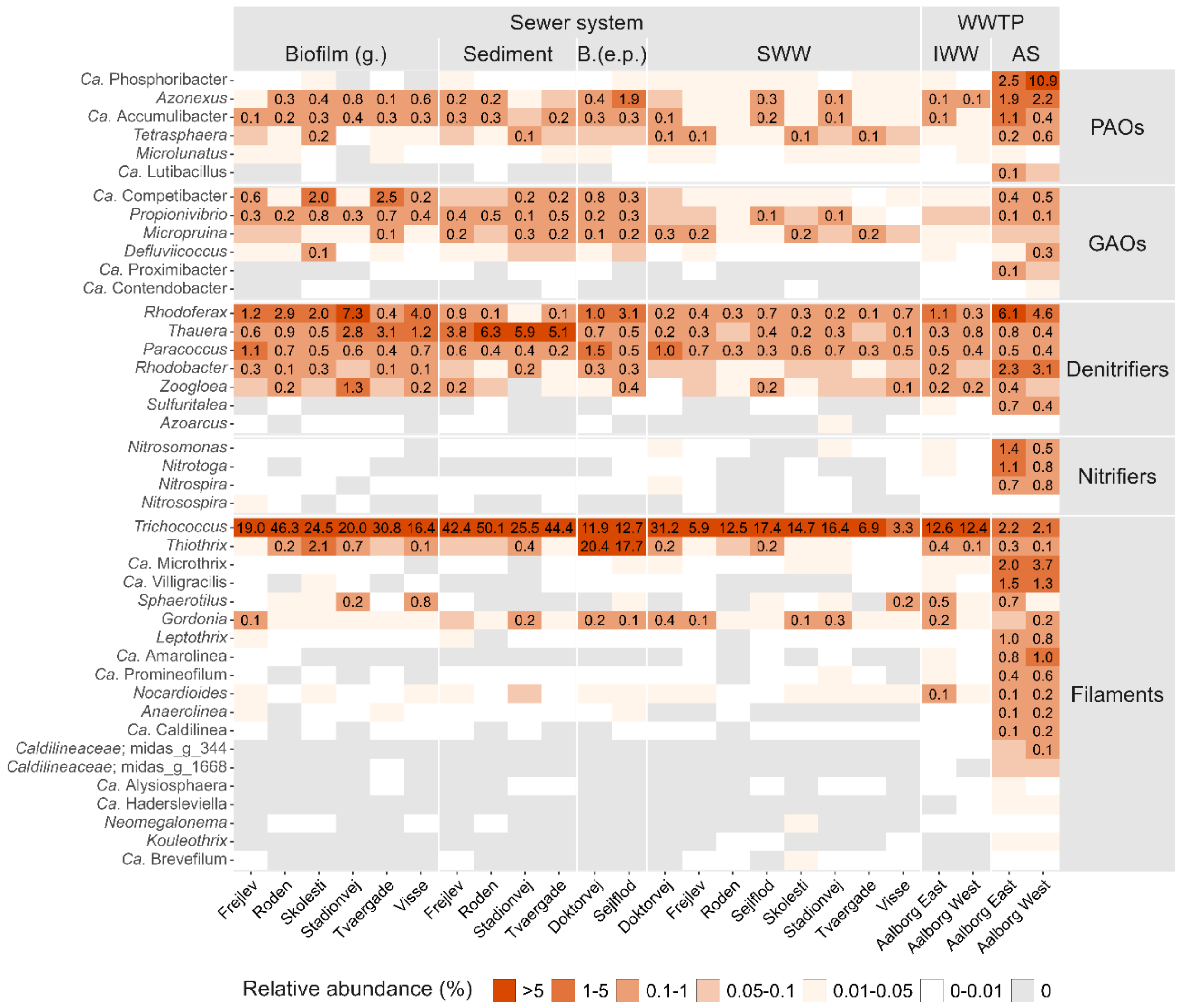

Figure S14: Abundance of bacteria (genus level) in each habitat and sample site grouped after known important functional guilds in AS. Abundances are shown with one decimal if ≥0.1% relative abundance. If no reads were found the abundance is 0%. Biofilm (e.p.) has been abbreviated B.(e.p.). Family names were provided for genera classified with MiDAS placeholder names.

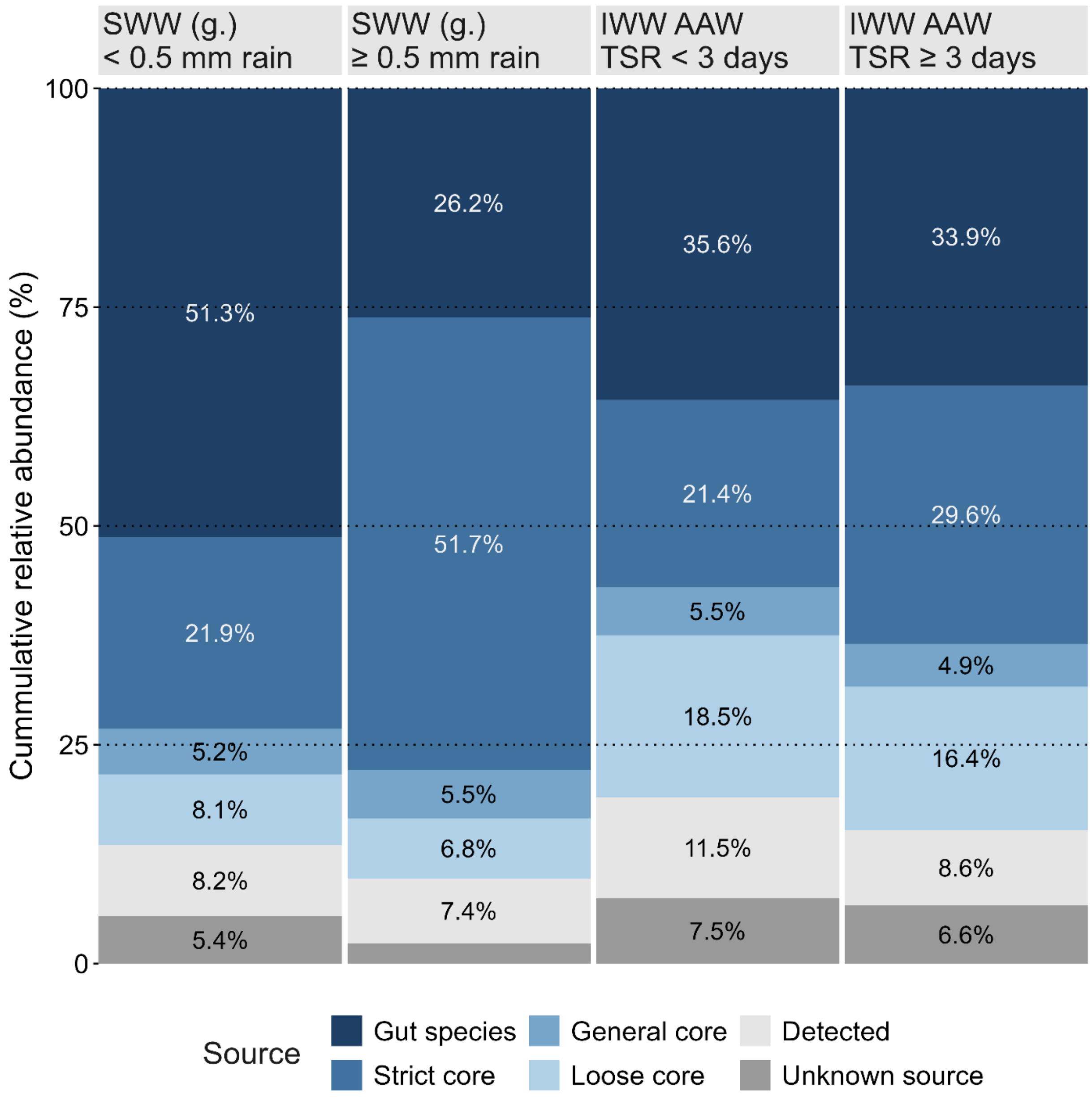

Figure S15: Cumulative abundance of the sewer core- and gut species grouped based on rainfall. SWW included all SWW samples in combined gravity sewers grouped depending on the rainfall within an hour before sampling (≥ 0.5 mm). All IWW samples were from Aalborg West (AAW) WWTP depending on the time since the rain (TSR) event (TSR ≥ 3 days) for the IWW samples.

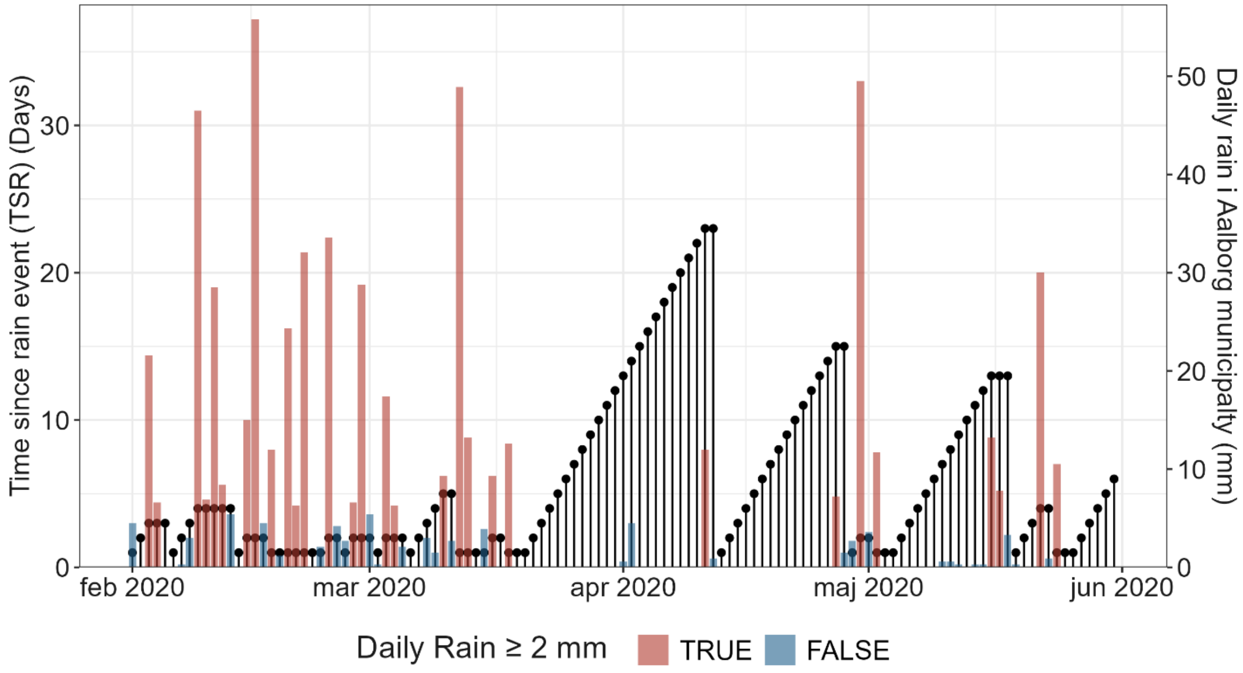

Figure S16: Time since rain events in Aalborg municipality. Time since rain event (TSR) is shown with black points and on the primary y-axis in days. The daily rainfall for Aalborg municipality is shown as red and blue bars and on the secondary y-axis in mm per day. Daily rain is colored based on detection of a rain event, corresponding to ≥ 2 mm during a day. Only a subset of the sample period is shown.

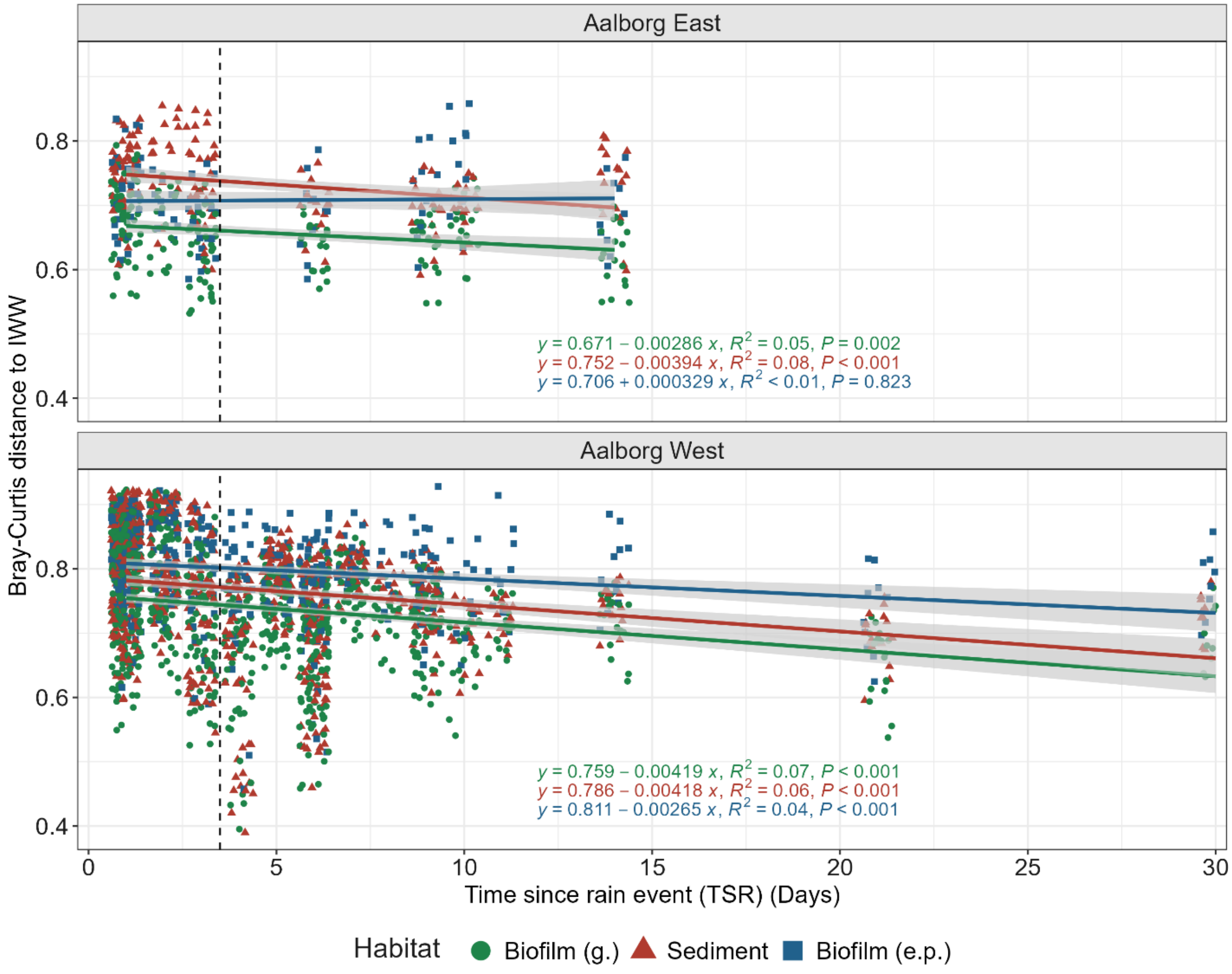

Figure S17: Bray-Curtis (BC) distance between IWW and sewer habitats as a function of time since rain (TSR) event for IWW from Aalborg East and West WWTP. A simple linear model was performed on the BC-distances between IWW samples and sediment; biofilm (g.); and biofilm (e.p.) samples to the TSR in days of the IWW sample. A stippled line marks TSR of 3.5 days. The equation for each habitat is shown on the plot where x-axis is the TSR and y-axis is the BC-distance as a function of TSR.
